# Supplementary material for: Approximate Solutions of a General Stochastic Velocity-Jump Model Subject to Discrete-Time Noisy Observations
Source: Bull Math Biol. 2025 Mar 25;87(5):57. doi: 10.1007/s11538-025-01437-x (PMC11937228; doi:10.1007/s11538-025-01437-x)
Supplement: Supplementary file 1 — (pdf 11258 KB) [file 11538_2025_1437_MOESM1_ESM.pdf]

Supplementary Information - Approximate solutions of a general stochastic velocity-jump model subject to discrete-time noisy observations

Arianna Ceccarelli[1*], Alexander P. Browning[1] and Ruth E. Baker[1]

[1]Mathematical Institute, University of Oxford, Oxford, OX2 6GG, Oxfordshire, UK.

*Corresponding author. E-mail: ceccarelli@maths.ox.ac.uk;

Contents

S1 Irreducibility hypothesis **2**

S2 Method to compute the initial probability distribution **2**

S3 Networks with parameters **3**

S4 Up-to-one-switch approximation for the probability distribution function of a noisy location increment **3**

S4.1 Computing $\mathbb{P}(\Delta y \,|\, W = 0, S_1 = s)$ . . . 5

S4.2 Computing $\mathbb{P}(W = 1 \,|\, S_1 = s_1, S_2 = s_2)$ . . . 5

S4.3 Computing $\mathbb{P}(\Delta x \,|\, W = 1, S_1 = s_1, S_2 = s_2)$ . . . 6

S4.4 Computing $\mathbb{P}(\Delta y \,|\, W = 1, S_1 = s_1, S_2 = s_2)$ . . . 7

S4.5 Error of the up-to-one-switch approximation . . . 8

S5 Up-to-two-switch approximation for the probability distribution function of a noisy location increment **8**

S5.1 Computing $\mathbb{P}(W = 2 \,|\, S_1 = s_1, S_2 = s_2, S_3 = s_3),\ s_3 \neq s_1$ . . . 10

S5.2 Computing $\mathbb{P}(\Delta x \,|\, W = 2, S_1 = s_1, S_2 = s_2, S_3 = s_3),\ s_3 \neq s_1$ . . . 11

S5.3 Computing $\mathbb{P}(\Delta y \,|\, W = 2, S_1 = s_1, S_2 = s_2, S_3 = s_3),\ s_3 \neq s_1$ . . . 14

S5.4 Computing $\mathbb{P}(W = 2 \,|\, S_1 = s_1, S_2 = s_2, S_3 = s_3)$ for $s_3 = s_1$ . . . 17

S5.5 Computing $\mathbb{P}(\Delta x \,|\, W = 2, S_1 = s_1, S_2 = s_2, S_3 = s_3)$ for $s_3 = s_1$ . . . 17

S5.6 Computing $\mathbb{P}(\Delta y \,|\, W = 2, S_1 = s_1, S_2 = s_2, S_3 = s_3)$ for $s_3 = s_1$ . . . 18

S5.7 Comparison of the error of the up-to-one-switch approximation with the error of the up-to-two-switch approximation . . . 19

S6 Up-to-one-switch approximation for the probability distribution function of a set of noisy subsequent location increments **19**

S6.1 Error of the up-to-one-switch approximation for the probability distribution function of two noisy subsequent location increments 23
S6.2 Extension to the up-to-two-switch approximation 23

S7 Comparison to the Fokker-Planck equation **25**

S1 Irreducibility hypothesis

We now present the irreducibility hypothesis for a CTMC. The Markov chain, or equivalently its matrix $\boldsymbol{Q}$, needs to be irreducible in order for $\mathbb{P}(s)$ to be constant at any time $t \geq 0$. $\boldsymbol{Q}$ is irreducible if and only if it leads to a single communicating class of states (Norris, 1998). In other words, for any states s and u there exists an integer $n \geq 0$ such that the probability of going from a state s to a state u in n switches is strictly positive, or equivalently, the probability of going from a state s to a state u after a time $t > 0$ is strictly positive (Norris, 1998). Proposition 2.59 in Liggett (2010) can be used to prove the existence of a stationary probability distribution, but it requires the chain to be recurrent in addition to it being irreducible. In fact, the irreducible chain considered is also recurrent, meaning that all its states are recurrent.

For an irreducible chain, Proposition 2.52 in Liggett (2010) states that a state is recurrent if and only if all states are recurrent. Moreover, if the chain is not recurrent it is transient, which would imply that the expected amount of time spent in u of a chain starting at s, denoted with $G(s, u)$, is finite for all $s, u \in \{1, 2, \ldots, n\}$. Hence, if by contradiction the chain was not recurrent, then, for a finite number of states, for any s, we would have $\sum_u G(s, u) < \infty$. But this cannot be true since

$$\sum_u G(s,u) = \sum_u \lim_{t\to\infty} \int_0^t p_y(s,u)\mathrm{d}t = \lim_{t\to\infty} \int_0^t \sum_u p_y(s,u)\mathrm{d}t = \lim_{t\to\infty} t = \infty.$$

Thus, we are guaranteed existence and uniqueness of a stationary probability distribution and therefore we can compute $\boldsymbol{\pi}$. We note that having sets of classes that do not communicate would lead to a set of disconnected models rather than a single one, which could be studied separately using the methods that follow.

S2 Method to compute the initial probability distribution

Here, we propose a method to compute the initial probability distribution $\boldsymbol{\pi}$ (Ross, 2014), assumed to be the stationary probability distribution of the Markov chain considered. The irreducibility assumption presented in Supplementary Information Section S1 guarantees the existence and uniqueness of $\boldsymbol{\pi}$.

For $\boldsymbol{Q}$ regular, $\boldsymbol{\pi}$ is a solution of the corresponding chemical master equation (Kuntz et al, 2021). In other words, we aim to find $\boldsymbol{\pi}$ such that

$$\boldsymbol{\pi}\boldsymbol{Q} = 0,$$

where

$$\boldsymbol{Q} = \begin{bmatrix} -\lambda_1 & \lambda_1 p_{12} & \lambda_1 p_{13} & \ldots & \lambda_1 p_{1n} \\ \lambda_2 p_{21} & -\lambda_2 & \lambda_2 p_{23} & \ldots & \lambda_2 p_{2n} \\ \lambda_3 p_{31} & \lambda_3 p_{32} & -\lambda_3 & \ldots & \lambda_3 p_{3n} \\ \vdots & \vdots & \vdots & \ddots & \vdots \\ \lambda_n p_{n1} & \lambda_n p_{n2} & \lambda_n p_{n3} & \ldots & -\lambda_n \end{bmatrix}.$$

We now compute an expression for a vector $\boldsymbol{\omega} = [\omega_1, \omega_2, \omega_3, \dots, \omega_n]^T \neq 0$ such that $\boldsymbol{Q}^T\boldsymbol{\omega} = \boldsymbol{0}$. The kernel of $\boldsymbol{Q}^T$ is not $\{\boldsymbol{0}\}$ since, by definition, the rows of $\boldsymbol{Q}$ are linearly dependent. Moreover, the irreducibility hypothesis guarantees that the dimension of the kernel is exactly one.

We can use inverse power method to find such eigenvector with zero eigenvalue (Ford, 2014). In particular, we obtain that for all states $s = 1, 2, \dots, n$,

$$\lambda_s\omega_s = \sum_{u\neq s} \lambda_u p_{us}\omega_u.$$

For all $s, u = 1, 2, \dots, n$, $u \neq s$, $\lambda_s > 0$ and $p_{su} \geq 0$ are non-negative coefficients. Hence, if there exists $\omega_s > 0$ then all the entries of $\boldsymbol{\omega}$ must be non-negative. Therefore, by defining $\Omega := \sum_{s=1,\dots,n} \omega_s$ and $p_s := \omega_i/\Omega$, we must have that $p_s \in [0, 1]$, for all states $s = 1, 2, \dots, n$.

S3 Networks with parameters

Figure S1 shows the full networks with parameters presented as examples. All the parameters are fixed, except the set of rates $\boldsymbol{\lambda} = [\lambda_1, \lambda_2, \dots, \lambda_n]$ which are often multiplied by a factor of 10 (or integers from 1 to 10) to obtain comparisons between data collection scenarios. The parameters are chosen to reflect the motion of molecular motors along microtubules since the movement is often captured in kymographs, which are intrinsically one-dimensional (see Figure 1**A**). The time step is fixed to be $\Delta t = 0.3$. Moreover, Maday et al (2014) estimate the velocities of molecular motors to reach the order of 1000, thus we define $v_F = 2000$ and $v_B = -1500$, and we set all stationary and pausing state velocities to zero. Finally, the noise is fixed to $\sigma = 50$, approximately an order of magnitude lower than the maximal exact increment magnitude, $v_F\Delta t = 600$.

S4 Up-to-one-switch approximation for the probability distribution function of a noisy location increment

Here, we compute explicitly all the results obtained in Section 3.1. The up-to-one-switch approximation of a single location increment is provided as a Python code in the function *approx_pdf_up_to_1_switch* in the file *functions.py*.

We now consider the up-to-one-switch approximation for the probability density function (PDF) of Δy, defined as

$$P_1(\Delta y) := \mathbb{P}(\Delta y \,|\, W = 0)\mathbb{P}(W = 0) + \mathbb{P}(\Delta y \,|\, W = 1)\mathbb{P}(W \geq 1). \tag{S1}$$

We make progress by further by conditioning each term in Equation (S1) on the state at the start of the interval, denoted as S_1. Namely, for $W = 0$, we see that

$$\mathbb{P}(\Delta y \,|\, W = 0)\mathbb{P}(W = 0) = \sum_{s=1}^{n} \mathbb{P}(\Delta y \,|\, W = 0, S_1 = s)\mathbb{P}(W = 0 \,|\, S_1 = s)\mathbb{P}(S_1 = s),$$

where $\mathbb{P}(S_1 = s) = p_s$ is given by the equilibrium assumption (Equation (2)). For $W = 0$, the amount of time spent in the first state is, trivially, given by Δt (see Figure 5 zero-switch case). Therefore we obtain

$$\mathbb{P}(W = 0 \,|\, S_1 = s) = \exp{(-\lambda_s\Delta t)}.$$

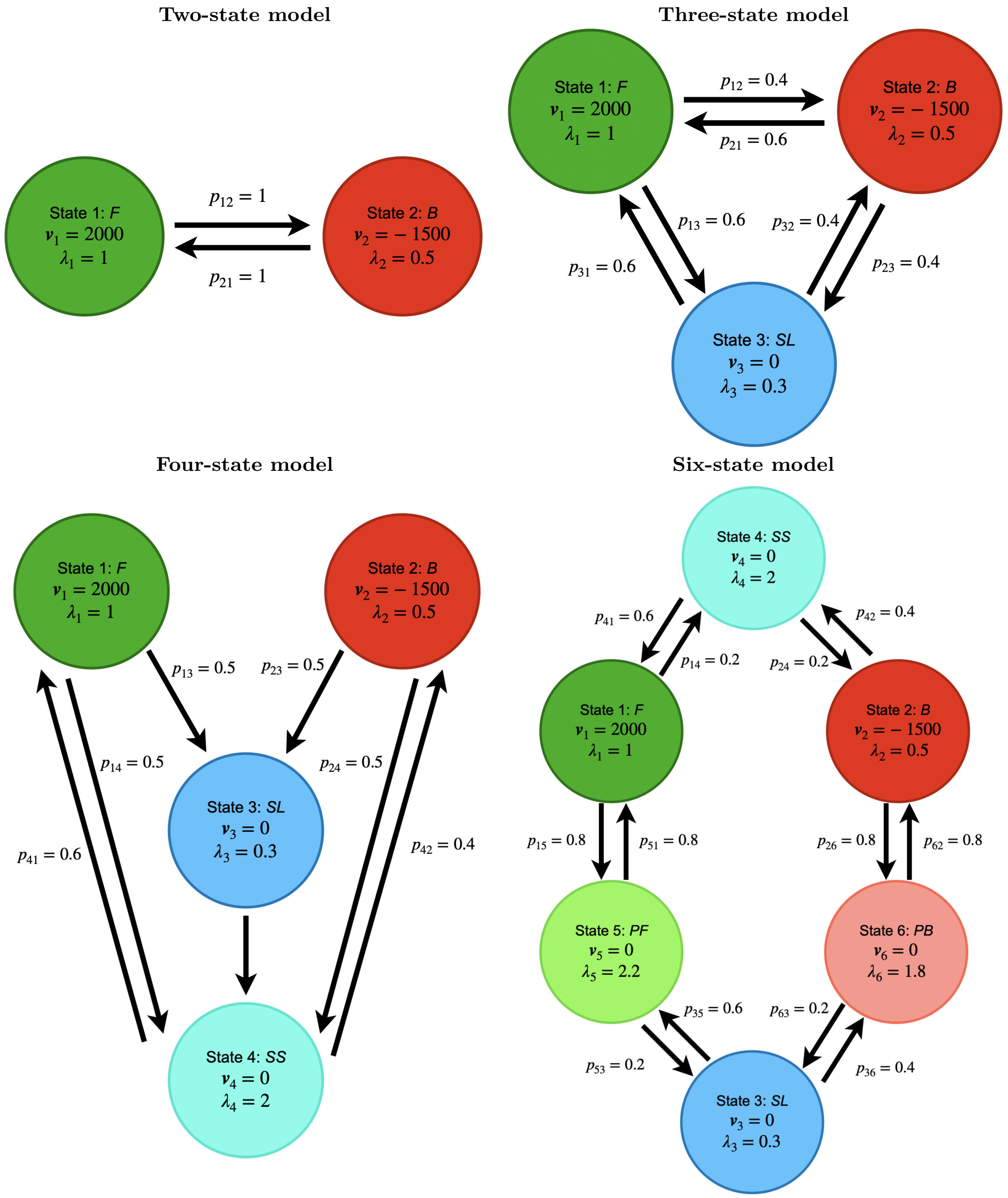

Fig. S1 Examples of n-state models with specified networks and parameters. A two-state, three-state, four-state and six-state model are presented. F stands for forward state, B for backward state, SL for stationary state with long average permanence, and SS for stationary state with short average permenence, PF for pause in forward movement and PB for pause in backward movement. Velocities and values of the transition matrix $P = (p_{ij})$ are fixed, while the switching rates λ are varied for testing purposes, but their ratios are fixed. Moreover, we fix $\Delta t = 0.3$ and $\sigma = 50$.

To compute $\mathbb{P}(\Delta y \,|\, W = 1)\mathbb{P}(W \geq 1)$, we condition on both the first state and the second state visited within the interval, S_1 and S_2 (see one-switch case in Figure 5). This yields

$$\begin{aligned}\mathbb{P}(\Delta y \,|\, W = 1)\mathbb{P}(W \geq 1) = \sum_{\substack{s_1 \\ s_2 \neq s_1}} & \mathbb{P}(\Delta y \,|\, W = 1, S_1 = s_1, S_2 = s_2) \\ & \times \mathbb{P}(W \geq 1 \,|\, S_1 = s_1, S_2 = s_2) \\ & \times \mathbb{P}(S_2 = s_2 \,|\, S_1 = s_1)\mathbb{P}(S_1 = s_1).\end{aligned}$$

By definition, $\mathbb{P}(S_2 = s_2 \,|\, S_1 = s_1) = p_{s_1 s_2}$ (Equation (1)), by the equilibrium assumption $\mathbb{P}(S_1 = s_1) = p_{s_1}$ (Equation (2)), and we obtain

$$\mathbb{P}(W \geq 1 \,|\, S_1 = s_1, S_2 = s_2) = \mathbb{P}(W \geq 1 \,|\, S_1 = s_1) = 1 - \exp(-\lambda_{s_1}\Delta t)$$

by considering that at least one switch occurs if $\tau_1 < \Delta t$.

S4.1 Computing $\mathbb{P}(\Delta y \,|\, W = 0, S_1 = s)$

For $W = 0$ and given the initial state of the interval S_1, we note that

$$(\Delta x \,|\, W = 0, S_1 = s) = v_s \Delta t.$$

Therefore,

$$\mathbb{P}(\Delta x \,|\, W = 0, S_1 = s) = \delta_{v_s \Delta t}(\Delta x),$$

where $\delta_{v_s \Delta t}$ denotes the Dirac delta function centered at $v_s \Delta t$. Adding the noise $\Delta\epsilon \sim \mathcal{N}(0, 2\sigma^2)$, we obtain

$$\mathbb{P}(\Delta y \,|\, W = 0, S_1 = s) = f_{\mathcal{N}(v_s\Delta t, 2\sigma^2)}(\Delta y),$$

where $f_{\mathcal{N}(v_s\Delta t, 2\sigma^2)}$ denotes the PDF of a normal distribution with mean $v_s \Delta t$ and variance $2\sigma^2$.

S4.2 Computing $\mathbb{P}(W = 1 \,|\, S_1 = s_1, S_2 = s_2)$

For $t_1 \in (0, \Delta t]$, we define

$$F_{s_1,s_2}(t_1) := \mathbb{P}(\tau_1 \leq t_1, \tau_1 > 0, \tau_2 > \Delta t - \tau_1 \,|\, S_1 = s_1, S_2 = s_2).$$

Then

$$G_{s_1,s_2}(t_1) := \mathbb{P}(\tau_1 \leq t_1 \,|\, 0 \leq \tau_1 \leq \Delta t, \tau_2 > \Delta t - \tau_1, S_1 = s_1, S_2 = s_2) = \frac{F_{s_1,s_2}(t)}{F_{s_1,s_2}(\Delta t)}.$$

We can compute F_{s_1,s_2} integrating the joint distribution $f_{s_1,s_2}(\tau_1, \tau_2)$ for $\tau_1 \in (0, t]$ and $\tau_2 > \Delta t - \tau_1$, thus

$$\begin{aligned} F_{s_1,s_2}(t_1) &:= \mathbb{P}(\tau_1 \leq t_1, \tau_1 > 0, \tau_2 > \Delta t - \tau_1 \,|\, S_1 = s_1, S_2 = s_2) \\ &= \int_0^{t_1} \left(\int_{\Delta t - \tau_1}^{\infty} f_{s_1,s_2}(\tau_1, \tau_2) \mathrm{d}\tau_2 \right) \mathrm{d}\tau_1. \end{aligned}$$

Note that $F_{s_1,s_2}(\Delta t) = \mathbb{P}(W = 1 \,|\, S_1 = s_1, S_2 = s_2)$. The joint distribution of two independent random variables is the product of the two distributions, and we note that $f_s(\tau) = \lambda_s e^{-\lambda_s t}$, which yields

$$f_{s_1,s_2}(\tau_1, \tau_2) = f_{s_1}(\tau_1) f_{s_2}(\tau_2) = \lambda_{s_1} \exp(-\lambda_{s_1}\tau_1)\, \lambda_{s_2} \exp(-\lambda_{s_2}\tau_2).$$

By substitution, we obtain

$$F_{s_1,s_2}(t_1) = \int_0^{t} \left(\int_{\Delta t - \tau_1}^{\infty} \lambda_{s_1} \exp(-\lambda_{s_1}\tau_1)\, \lambda_{s_2} \exp(-\lambda_{s_2}\tau_2)\, \mathrm{d}\tau_2 \right) \mathrm{d}\tau_1,$$

thus,

$$F_{s_1,s_2}(t) = \begin{cases} \lambda_{s_1} \exp(-\lambda_{s_1}\Delta t)\, t & \text{if } \lambda_{s_1} = \lambda_{s_2}, \\ \dfrac{\lambda_{s_1} \exp(-\lambda_{s_2}\Delta t)}{-\lambda_{s_1} + \lambda_{s_2}} \left(\exp((-\lambda_{s_1} + \lambda_{s_2})t) - 1\right) & \text{if } \lambda_{s_1} \neq \lambda_{s_2}. \end{cases}$$

It follows that

$$G_{s_1,s_2}(t) = \begin{cases} \dfrac{t}{\Delta t} & \text{if } \lambda_{s_1} = \lambda_{s_2}, \\ \dfrac{\exp\left((-\lambda_{s_1} + \lambda_{s_2})t\right) - 1}{\exp\left((-\lambda_{s_1} + \lambda_{s_2})\Delta t\right) - 1} & \text{if } \lambda_{s_1} \neq \lambda_{s_2}. \end{cases}$$

Finally, we note that $\mathbb{P}(W = 1 \mid S_1 = s_1, S_2 = s_2) = F_{s_1,s_2}(\Delta t)$.

S4.3 Computing $\mathbb{P}(\Delta x \mid W = 1, S_1 = s_1, S_2 = s_2)$

For fixed $S_1 = s_1$, $S_2 = s_2$ and a time τ_1 spent in state S_1 during the time interval considered, we get

$$(\Delta x \mid W = 1, S_1 = s_1, S_2 = s_2) = v_{s_1}\tau_1 + v_{s_2}(\Delta t - \tau_1).$$

We define

$$h_{s_1,s_2}(\tau_1) := v_{s_1}\tau_1 + v_{s_2}(\Delta t - \tau_1).$$

We note that $h_{s_1,s_2}(t)$ is monotonically increasing if $v_{s_1} - v_{s_2} > 0$, and decreasing if $v_{s_1} - v_{s_2} < 0$.

If $v_{s_1} = v_{s_2}$, then the velocity is constant for the whole interval; thus the exact location increment is known $\Delta x = h_{s_1,s_2}(\Delta t)$ and

$$\mathbb{P}(\Delta y \mid W = 1, S_1 = s_1, S_2 = s_2) = \mathbb{P}(\Delta y \mid W = 0, S_1 = s_1) = f_{\mathcal{N}(v_{s_1}\Delta t, 2\sigma^2)}(\Delta y).$$

Otherwise, the exact increment Δx is determined by the time of the switch, and we compute

$$\begin{aligned} g_{s_1,s_2}(t_1) := \mathbb{P}(t_1 \mid W = 1, S_1 = s_1, S_2 = s_2) &= \frac{\mathrm{d}}{\mathrm{d}t} G_{s_1,s_2}(t_1) \\ &= \begin{cases} \dfrac{1}{\Delta t} & \text{if } \lambda_{s_1} = \lambda_{s_2}, \\ (-\lambda_{s_1} + \lambda_{s_2}) \dfrac{\exp\left((-\lambda_{s_1} + \lambda_{s_2})t\right) - 1}{\exp\left((-\lambda_{s_1} + \lambda_{s_2})\Delta t\right) - 1} & \text{if } \lambda_{s_1} \neq \lambda_{s_2}. \end{cases} \end{aligned}$$

Since $h_{s_1,s_2} : \mathbb{R} \to \mathbb{R}$ is a monotonic function and $\Delta x = h_{s_1,s_2}(t)$, the density function of Δx is

$$\begin{aligned} \tilde{g}_{s_1,s_2}(\Delta x) &= \mathbb{P}(\Delta x \mid W = 1, S_1 = s_1, S_2 = s_2) \\ &= g_{s_1,s_2}\left(h^{-1}_{s_1,s_2}(\Delta x)\right) \left| \frac{\mathrm{d}}{\mathrm{d}(\Delta x)} h^{-1}_{s_1,s_2}(\Delta x) \right|. \end{aligned}$$

Hence, we compute t in terms of Δx as

$$t = h^{-1}_{s_1,s_2}(\Delta x) = \frac{\Delta x - v_{s_2}\Delta t}{v_{s_1} - v_{s_2}}$$

and its derivative

$$\frac{\mathrm{d}}{\mathrm{d}(\Delta x)} h^{-1}_{s_1,s_2}(\Delta x) = \frac{1}{v_{s_1} - v_{s_2}}.$$

Hence,

$$\left| \frac{\mathrm{d}}{\mathrm{d}(\Delta x)} h^{-1}_{s_1,s_2}(\Delta x) \right| = \frac{1}{|v_{s_1} - v_{s_2}|}.$$

S4.4 Computing $\mathbb{P}(\Delta y \,|\, W = 1, S_1 = s_1, S_2 = s_2)$

Now, we incorporate noise

$$\tilde{f}_{s_1,s_2}(\Delta y) := \mathbb{P}(\Delta y \,|\, W = 1, S_1 = s_1, S_2 = s_2) \\ = \int_a^b \tilde{g}_{s_1,s_2}(\Delta x) f_{\mathcal{N}(0,2\sigma^2)}(\Delta y - \Delta x)\mathrm{d}(\Delta x),$$

where

$$a = a_{s_1,s_2} := \min\{h_{s_1,s_2}(0), h_{s_1,s_2}(\Delta t)\},$$
$$b = b_{s_1,s_2} := \max\{h_{s_1,s_2}(0), h_{s_1,s_2}(\Delta t)\},$$

and

$$f_{\mathcal{N}(0,2\sigma^2)}(\Delta\epsilon) := \frac{1}{\sqrt{2}\sigma\sqrt{2\pi}} \exp\left(-\frac{1}{2}\frac{(\Delta\epsilon)^2}{2\sigma^2}\right) = \frac{1}{2\sigma\sqrt{\pi}} \exp\left(-\frac{(\Delta\epsilon)^2}{4\sigma^2}\right).$$

First, we consider the case $\lambda_{s_1} \neq \lambda_{s_2}$. Then,

$$\tilde{g}_{s_1,s_2}(\Delta x) = \frac{-\lambda_{s_1} + \lambda_{s_2}}{|v_{s_1} - v_{s_2}|} \frac{\exp\left((-\lambda_{s_1} + \lambda_{s_2}) h^{-1}_{s_1,s_2}(\Delta x)\right) - 1}{\exp\left((-\lambda_{s_1} + \lambda_{s_2})\Delta t\right) - 1}.$$

To obtain $\tilde{f}_{s_1,s_2}(\Delta y)$ we integrate over Δx. We note that

$$\int_a^b \exp\left(-c(\Delta x)^2 + r\Delta x + \hat{r}\right)\mathrm{d}(\Delta x) = \frac{\sqrt{\pi}}{2\sqrt{c}} \exp\left(\frac{r^2}{4c} + \hat{r}\right) \left[\mathrm{erf}\left(\frac{2c\Delta x - r}{2\sqrt{c}}\right)\right]_{\Delta x = a}^{\Delta x = b},$$

where erf is the error function, and write

$$\tilde{f}_{s_1,s_2}(\Delta y) = k \left[\mathrm{erf}\left(\frac{2c\Delta x - r}{2\sqrt{c}}\right)\right]_{\Delta x = a}^{\Delta x = b}.$$

Here, a and b are as previously defined,

$$c := \frac{1}{4\sigma^2},$$

while, r, $\hat{r}$ and k vary depending on the states s_1, s_2 and on Δy:

$$r = r_{s_1,s_2}(\Delta y) := 2c\Delta y + \frac{-\lambda_{s_1} + \lambda_{s_2}}{v_{s_1} - v_{s_2}},$$

$$\hat{r} = \hat{r}_{s_1,s_2}(\Delta y) := -c(\Delta y)^2 + \frac{-\lambda_{s_1} + \lambda_{s_2}}{|v_{s_1} - v_{s_2}|} |v_{s_2}| \Delta t,$$

and

$$k = k_{s_1,s_2} := \frac{-\lambda_{s_1} + \lambda_{s_2}}{2|v_{s_1} - v_{s_2}|} \frac{\exp\left(\frac{r^2}{4c} + \hat{r}\right)}{\exp\left((-\lambda_{s_1} + \lambda_{s_2})\Delta t\right) - 1}.$$

If $\lambda_{s_1} = \lambda_{s_2}$, then

$$\tilde{g}_{s_1,s_2}(\Delta x) = \frac{1}{|v_{s_1} - v_{s_2}|\Delta t},$$

and we note that here $\tilde{g}_{s_1,s_2}(\Delta x)$ is the length of the interval $[a, b]$, as previously defined. Thus

$$\tilde{g}_{s_1,s_2}(\Delta x) = f_{\mathcal{U}_{(a,b)}}(\Delta x),$$

where $f_{\mathcal{U}_{(a,b)}}$ denotes the PDF of a uniform distribution in the interval $[a, b]$.

Therefore,

$$\tilde{f}_{s_1,s_2}(\Delta y) = \int_a^b f_{\mathcal{U}(a,b)}(\Delta x) f_{\mathcal{N}(0,2\sigma^2)}(\Delta y - \Delta x)\mathrm{d}(\Delta x) = \frac{1}{b-a}\int_{\Delta y - a}^{\Delta y - b} -f_{\mathcal{N}(0,2\sigma^2)}(w)\mathrm{d}w,$$

where $f_{\mathcal{U}(a,b)} = 1/(b-a)$ is the PDF of the uniform distribution in $[a,b]$ and the change of variable $W = \Delta y - \Delta x$. Hence,

$$\tilde{f}_{s_1,s_2}(\Delta y) = \frac{1}{b-a}\left(F_{\mathcal{N}(0,2\sigma^2)}(\Delta y - a) - F_{\mathcal{N}(0,2\sigma^2)}(\Delta y - b)\right),$$

where $F_{\mathcal{N}(0,2\sigma^2)}$ represents the CDF of the normal distribution with mean $\mu = 0$ and variance $\hat{\sigma}^2 = 2\sigma^2$, defined as

$$F_{\mathcal{N}(\mu,\hat{\sigma})}(w) = \frac{1}{2}\left[1 + \operatorname{erf}\left(\frac{w-\mu}{\hat{\sigma}\sqrt{2}}\right)\right].$$

We note that $\tilde{f}_{s_1,s_2}(\Delta y)$ for $\lambda_1 = \lambda_2$ can also be obtained by taking the limit for $\lambda_1 - \lambda_2 \to 0$ of the function computed for $\lambda_1 \neq \lambda_2$.

S4.5 Error of the up-to-one-switch approximation

Figure S2 shows the error of the up-to-one-switch approximation, $P_1(\Delta y) - P(\Delta y)$, for the four-state model and the six-state model.

S5 Up-to-two-switch approximation for the probability distribution function of a noisy location increment

Here, we compute explicitly all the results obtained in Section 3.2. The up-to-two-switch approximation of a single location increment is provided as a Python code in the function *approx_pdf_up_to_2_switch* in the file *functions.py*.

We now consider the up-to-two-switch approximation for the PDF of Δy defined as

$$P_2(\Delta y) := \mathbb{P}(\Delta y \,|\, W = 0)\mathbb{P}(W = 0) + \mathbb{P}(\Delta y \,|\, W = 1)\mathbb{P}(W = 1) + \mathbb{P}(\Delta y \,|\, W = 2)\mathbb{P}(W \geq 2).$$

We have already computed $\mathbb{P}(\Delta y \,|\, W = 0)\mathbb{P}(W = 0)$ and $\mathbb{P}(\Delta y \,|\, W = 1)$ by conditioning on the states attained, therefore we need to compute $\mathbb{P}(W = 1)$ and $\mathbb{P}(\Delta y \,|\, W = 2)\mathbb{P}(W \geq 2)$.

We note that

$$\mathbb{P}(W \geq 2) = 1 - \mathbb{P}(W = 0) - \mathbb{P}(W = 1),$$

which also holds after conditioning on the states attained. And in particular,

$$\begin{aligned}\mathbb{P}(W \geq 2 \,|\, S_1 = s_1, S_2 = s_2, S_3 = s_3) &= 1 - \mathbb{P}(W = 0 \,|\, S_1 = s_1) - \mathbb{P}(W = 1 \,|\, S_1 = s_1, S_2 = s_2) \\ &= \mathbb{P}(W \geq 2 \,|\, S_1 = s_1, S_2 = s_2),\end{aligned} \quad \text{(S2)}$$

where $\mathbb{P}(W = 0 \,|\, S_1 = s_1)$ and $\mathbb{P}(W = 1 \,|\, S_1 = s_1, S_2 = s_2)$ have been computed in the previous section.

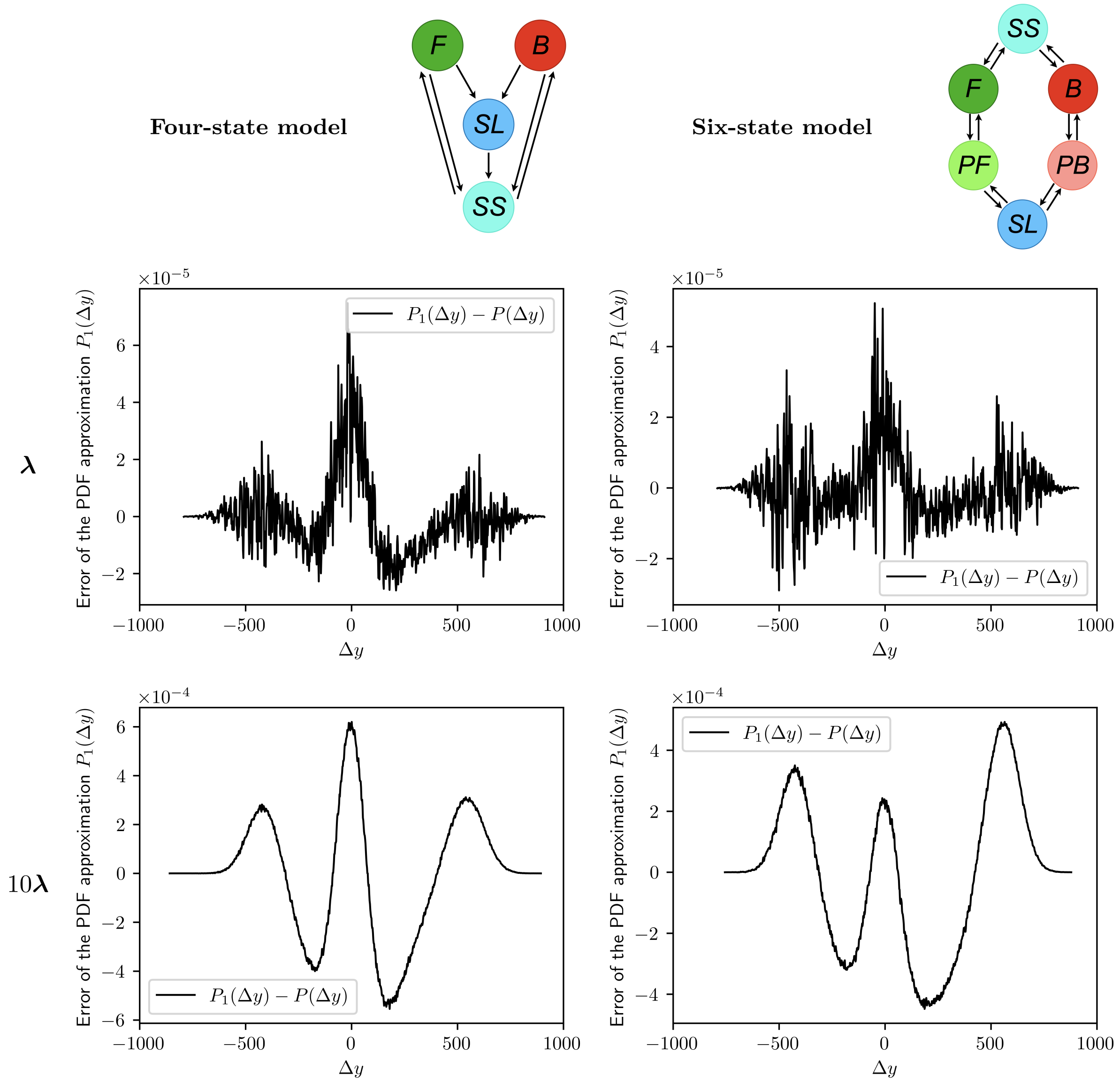

Fig. S2 The error of the approximation $P_1(\Delta y)$, defined as $P_1(\Delta y) - P(\Delta y)$ where $P(\Delta y)$ is the empirical PDF for Δy is shown. The left plots are obtained from a four-state model while the right ones from a six-state model. The top plots are obtained using the parameters as specified in the Supplementary Information Figure S1, while the bottom ones use the same parameters except the rates which are multiplied by 10.

Moreover, as in the one-switch case, we need to condition on the states. For two switches, we have three subsequent states to consider

$$\begin{aligned}\mathbb{P}(\Delta y \,|\, W = 2)\mathbb{P}(W \geq 2) = \sum_{s_1} \sum_{s_2 \neq s_1} \sum_{s_3 \neq s_2} &\mathbb{P}(\Delta y \,|\, W = 2, S_1 = s_1, S_2 = s_2, S_3 = s_3) \\ &\times \mathbb{P}(W \geq 2 \,|\, S_1 = s_1, S_2 = s_2, S_3 = s_3) \\ &\times \mathbb{P}(S_3 = s_3 \,|\, S_1 = s_1, S_2 = s_2) \\ &\times \mathbb{P}(S_2 = s_2 \,|\, S_1 = s_1)\mathbb{P}(S_1 = s_1),\end{aligned}$$

where $\mathbb{P}(S_3 = s_3 \,|\, S_1 = s_1, S_2 = s_2) = \mathbb{P}(S_3 = s_3 \,|\, S_2 = s_2) = p_{s_2,s_3}$, $\mathbb{P}(S_2 = s_2 \,|\, S_1 = s_1) = p_{s_1,s_2}$ and $\mathbb{P}(S_1 = s_1) = p_{s_1}$. Hence, we only need to compute $\mathbb{P}(\Delta y \,|\, W = 2, S_1 = s_1, S_2 = s_2, S_3 = s_3)$ and, as before, we start by computing $\mathbb{P}(W = 2 \,|\, S_1 = s_1, S_2 = s_2, S_3 = s_3)$.

S5.1 Computing $\mathbb{P}(W = 2 \,|\, S_1 = s_1, S_2 = s_2, S_3 = s_3),\ s_3 \neq s_1$

Here we compute $\mathbb{P}(W = 2 \,|\, S_1 = s_1, S_2 = s_2, S_3 = s_3)$ when $s_3 \neq s_1$. We consider the case $s_3 = s_1$ separately, in Section S5.4. Moreover, we assume that the velocities v_{s_i} are all distinct and the rates λ_{s_i} are all distinct for $i = 1, 2, 3$. We denote τ_i the time spent in state S_i during the Δt time interval considered. For $W = 2$, we need to condition on the three subsequent states attained (S_1, S_2, S_3) to have all the necessary information to compute $\mathbb{P}(W = 2 \,|\, S_1 = s_1, S_2 = s_2, S_3 = s_3)$. The number of switches is $W = 2$ if and only if $0 < \tau_1 \leq \Delta t$, $0 < \tau_2 \leq \Delta t - \tau_1$ and $\tau_3 \geq \Delta t - \tau_1 - \tau_2$.

For $t_1, t_2 \in (0, \Delta t]$ with $t_2 \leq \Delta t - t_1$, we define

$$\begin{aligned} F_{s_1,s_2,s_3}(t_1,t_2) &:= \mathbb{P}\left(\begin{matrix} \tau_1 \leq t_1, \tau_2 \leq t_2, \tau_1 > 0, \\ 0 < \tau_2 \leq \Delta t - \tau_1, \tau_3 > \Delta t - \tau_1 - \tau_2 \end{matrix} \,\middle|\, S_1 = s_1, S_2 = s_2, S_3 = s_3 \right) \\ &= \mathbb{P}(0 < \tau_1 \leq t_1, 0 < \tau_2 \leq t_2, \tau_3 > \Delta t - \tau_1 - \tau_2 \,|\, S_1 = s_1, S_2 = s_2, S_3 = s_3), \end{aligned}$$

since $t_2 \leq \Delta t - t_1 \leq \Delta t - \tau_1$. We can compute F_{s_1,s_2,s_3} by integrating the joint distribution $f_{s_1,s_2,s_3}(\tau_1,\tau_2,\tau_3)$ for $\tau_1 \in (0, t_1]$ and $\tau_2 \in (0, t_2]$ and $\tau_3 > \Delta t - \tau_1 - \tau_2$, thus

$$F_{s_1,s_2,s_3}(t_1,t_2) := \int_0^{t_1} \left(\int_0^{t_2} \left(\int_{\Delta t - \tau_1 - \tau_2}^{\infty} f_{s_1,s_2,s_3}(\tau_1,\tau_2,\tau_3) \mathrm{d}\tau_3 \right) \mathrm{d}\tau_2 \right) \mathrm{d}\tau_1.$$

The joint distribution of three independent random variables is the product of the three distributions, and we note that $f_s(\tau) = \lambda_s e^{-\lambda_s \tau}$; hence,

$$\begin{aligned} f_{s_1,s_2,s_3}(\tau_1,\tau_2,\tau_3) &= f_{s_1}(\tau_1) f_{s_2}(\tau_2) f_{s_2}(\tau_3) \\ &= \lambda_{s_1} \exp\left(-\lambda_{s_1}\tau_1\right) \lambda_{s_2} \exp\left(-\lambda_{s_2}\tau_2\right) \lambda_{s_3} \exp\left(-\lambda_{s_3}\tau_3\right). \end{aligned}$$

Hence, in the assumption that λ_{s_i} are all distinct, we obtain

$$F_{s_1,s_2,s_3}(t_1,t_2) = \lambda_{s_1}\lambda_{s_2} \exp\left(-\lambda_{s_3}\Delta t\right) \frac{\exp\left((-\lambda_{s_2}+\lambda_{s_3})t_2\right) - 1}{-\lambda_{s_2}+\lambda_{s_3}} \frac{\exp\left((-\lambda_{s_1}+\lambda_{s_3})t_1\right) - 1}{-\lambda_{s_1}+\lambda_{s_3}}.$$

We define

$$G_{s_1,s_2,s_3}(t_1,t_2) := \mathbb{P}\left(\tau_1 \leq t_1, \tau_2 \leq t_2 \,\middle|\, \begin{matrix} 0 < \tau_1 \leq \Delta t, 0 < \tau_2 \leq \Delta t - \tau_1, \tau_3 > \Delta t - \tau_1 - \tau_2, \\ S_1 = s_1, S_2 = s_2, S_3 = s_3 \end{matrix} \right),$$

which can be computed as

$$G_{s_1,s_2,s_3}(t_1,t_2) := \frac{F_{s_1,s_2,s_3}(t_1,t_2)}{\mathbb{P}\left(0 < \tau_1 \leq \Delta t, 0 < \tau_2 \leq \Delta t - \tau_1, \tau_3 > \Delta t - \tau_1 - \tau_2 \,|\, S_1 = s_1, S_2 = s_2, S_3 = s_3\right)},$$

where

$$\begin{aligned} &\mathbb{P}(0 < \tau_1 \leq \Delta t, 0 < \tau_1 \leq \Delta t - \tau_2, \tau_3 > \Delta t - \tau_1 - \tau_2 \,|\, S_1 = s_1, S_2 = s_2, S_3 = s_3) \\ &= \int_0^{\Delta t} \left(\int_0^{\Delta t - \tau_1} \left(\int_{\Delta t - \tau_1 - \tau_2}^{\infty} f_{s_1,s_2,s_3}(\tau_1,\tau_2,\tau_3) \mathrm{d}\tau_3 \right) \mathrm{d}\tau_2 \right) \mathrm{d}\tau_1 \\ &= \frac{\lambda_{s_1}\lambda_{s_2}}{-\lambda_{s_2}+\lambda_{s_3}} \left(\frac{\exp\left(-\lambda_{s_1}\Delta t\right) - \exp\left(-\lambda_{s_2}\Delta t\right)}{-\lambda_{s_1}+\lambda_{s_2}} - \frac{\exp\left(-\lambda_{s_1}\Delta t\right) - \exp\left(-\lambda_{s_3}\Delta t\right)}{-\lambda_{s_1}+\lambda_{s_3}} \right). \end{aligned}$$

Therefore, simplifying we obtain

$$G_{s_1,s_2,s_3}(t_1,t_2) = \frac{\exp\left(-\lambda_{s_3}\Delta t\right)\left(\exp\left((-\lambda_{s_2}+\lambda_{s_3})t_2\right)-1\right)\left(\exp\left((-\lambda_{s_1}+\lambda_{s_3})t_1\right)-1\right)}{\left(\frac{\exp\left(-\lambda_{s_1}\Delta t\right)-\exp\left(-\lambda_{s_2}\Delta t\right)}{-\lambda_{s_1}+\lambda_{s_2}} - \frac{\exp\left(-\lambda_{s_1}\Delta t\right)-\exp\left(-\lambda_{s_3}\Delta t\right)}{-\lambda_{s_1}+\lambda_{s_3}}\right)\left(-\lambda_{s_1}+\lambda_{s_3}\right)}.$$

Finally, we note that

$$\mathbb{P}(W=2 \mid S_1=s_1, S_2=s_2, S_3=s_3) = F_{s_1,s_2,s_3}(\Delta t, \Delta t).$$

S5.2 Computing $\mathbb{P}(\Delta x \mid W=2, S_1=s_1, S_2=s_2, S_3=s_3),\ s_3 \neq s_1$

From the CDF $G_{s_1,s_2,s_3}(t_1,t_2)$ we can obtain the PDF

$$g_{s_1,s_2,s_3}(t_1,t_2) := \mathbb{P}(t_1,t_2 \mid W=2, S_1=s_1, S_2=s_2, S_3=s_3) = \frac{\partial^2}{\partial t_1 \partial t_2} G_{s_1,s_2,s_3}(t_1,t_2).$$

Simplifying we obtain

$$g_{s_1,s_2,s_3}(t_1,t_2) := \frac{N(t_1,t_2)}{D},$$

where we define $D := D_{1,2} + D_{2,3} + D_{3,1}$,

$$D_{i,j} := (\lambda_{s^{(i)}} - \lambda_{s^{(j)}}) \exp\left(\Delta t(\lambda_{s^{(i)}} + \lambda_{s^{(j)}})\right),$$

and

$$N(t_1,t_2) := D_{1,2} \cdot (\lambda_{s_3} - \lambda_{s_1}) \exp\left(t_1(\lambda_{s_3} - \lambda_{s_1})\right) \cdot (\lambda_{s_3} - \lambda_{s_2}) \exp\left(t_2(\lambda_{s_3} - \lambda_{s_2})\right).$$

We define

$$\begin{aligned}
\tilde{g}_{s_1,s_2,s_3}(\Delta x) &:= \mathbb{P}(\Delta x \mid W=2, S_1=s_1, S_2=s_2, S_3=s_3) = \\
&= \int_0^{\Delta t} \Big(\int_0^{\Delta t - t_1} \mathbb{P}(\Delta x \mid \tau_1 = t_1, \tau_2 = t_2, W=2, S_1=s_1, S_2=s_2, S_3=s_3) \\
&\qquad \times \mathbb{P}(t_1,t_2 \mid W=2, S_1=s_1, S_2=s_2, S_3=s_3) \mathrm{d}t_2 \Big) \mathrm{d}t_1 \\
&= \int_0^{\Delta t} \left(\int_0^{\Delta t - t_1} \delta\left(v_{s_1}t_1 + v_{s_2}t_2 + v_{s_3}(\Delta t - t_1 - t_2) - \Delta x\right) g_{s_1,s_2,s_3}(t_1,t_2) \mathrm{d}t_2 \right) \mathrm{d}t_1.
\end{aligned}$$

Now, we use the sifting property of the Dirac delta function: for any continuous function f,

$$\int_{-\infty}^{\infty} \delta(x-s) f(s) \mathrm{d}s = f(x).$$

Since the Dirac delta function is evaluated at

$$[(v_{s_1} - v_{s_3})t_1 + v_{s_3}\Delta t - \Delta x] - [(v_{s_3} - v_{s_2})t_2],$$

for $v_{s_3} \neq v_{s_2}$, we use the changes of variable $x = x(t_1) := (v_{s_1} - v_{s_3})t_1 + v_{s_3}\Delta t - \Delta x$ and $z = z(t_2) := (v_{s_3} - v_{s_2})t_2$, and we obtain

$$\begin{aligned}&\int_0^{\Delta t - t_1} \delta\left(v_{s_1}t_1 + v_{s_2}t_2 + v_{s_3}(\Delta t - t_1 - t_2) - \Delta x\right) g_{s_1,s_2,s_3}(t_1,t_2)\mathrm{d}t_2 \\ &= \int_{z^{-1}(0)}^{z^{-1}(\Delta t - t_1)} \delta(x-z)\ g_{s_1,s_2,s_3}\left(t_1, \frac{z}{v_{s_3}-v_{s_2}}\right)\frac{1}{v_{s_3}-v_{s_2}}\mathrm{d}z.\end{aligned}$$

We note that $z^{-1}(0) = 0$ and $z^{-1}(\Delta t - t_1) = (\Delta t - t_1)/(v_{s_3} - v_{s_2})$ and for all $t_1 \in [0, \Delta t]$

$$\begin{cases} z^{-1}(0) \leq z^{-1}(\Delta t - t_1) & \text{if } v_{s_3} - v_{s_2} > 0, \\ z^{-1}(0) \geq z^{-1}(\Delta t - t_1) & \text{if } v_{s_3} - v_{s_2} < 0. \end{cases}$$

Hence, $\operatorname{sign}\left(z^{-1}(\Delta t - t_1) - z^{-1}(0)\right) = \operatorname{sign}\left(v_{s_3} - v_{s_2}\right)$. Moreover, since $g_{s_1,s_2,s_3}(t_1,t_2)$ is only defined for $t_1 \in [0,\Delta t]$ and $t_2 \in [0, \Delta t - t_1]$, we extend its definition to $\mathbb{R}$ using the function

$$\bar{g}_{s_1,s_2,s_3}(t_1,t_2) := \begin{cases} g_{s_1,s_2,s_3}(t_1,t_2) & \text{if } t_1 \in [0,\Delta t], t_2 \in [0,\Delta t - t_1], \\ 0 & \text{otherwise.} \end{cases}$$

Hence, we write the integral

$$\begin{aligned}&\int_{z^{-1}(0)}^{z^{-1}(\Delta t - t_1)} \delta(x-z)\ g_{s_1,s_2,s_3}\left(t_1, \frac{z}{v_{s_3}-v_{s_2}}\right)\frac{1}{v_{s_3}-v_{s_2}}\mathrm{d}z \\ &= \frac{\operatorname{sign}\left(v_{s_3}-v_{s_2}\right)}{v_{s_3}-v_{s_2}} \int_{-\infty}^{\infty} \delta(x-z)\ \bar{g}_{s_1,s_2,s_3}\left(t_1, \frac{z}{v_{s_3}-v_{s_2}}\right)\mathrm{d}z \\ &= \frac{1}{|v_{s_3}-v_{s_2}|}\ \bar{g}_{s_1,s_2,s_3}\left(t_1, \frac{x}{v_{s_3}-v_{s_2}}\right) \\ &= \frac{1}{|v_{s_3}-v_{s_2}|}\ \bar{g}_{s_1,s_2,s_3}\left(t_1, \frac{(v_{s_1}-v_{s_3})t_1 + v_{s_3}\Delta t - \Delta x}{v_{s_3}-v_{s_2}}\right).\end{aligned}$$

We also see that

$$g_{s_1,s_2,s_3}\left(t_1, \frac{(v_{s_1}-v_{s_3})t_1 + v_{s_3}\Delta t - \Delta x}{v_{s_3}-v_{s_2}}\right) = \frac{1}{D}\cdot N\left(t_1, \frac{(v_{s_1}-v_{s_3})t_1 + v_{s_3}\Delta t - \Delta x}{v_{s_3}-v_{s_2}}\right),$$

where we define

$$N(t_1,t_2) := (\lambda_{s_1} - \lambda_{s_2})\exp\left(\Delta t(\lambda_{s_1}+\lambda_{s_2})\right)(\lambda_{s_3}-\lambda_{s_1})\exp\left(t_1(\lambda_{s_3}-\lambda_{s_1})\right)(\lambda_{s_3}-\lambda_{s_2})\exp\left(t_2(\lambda_{s_3}-\lambda_{s_2})\right).$$

Thus, by substitution we get

$$\begin{aligned}N\left(t_1, \frac{(v_{s_1}-v_{s_3})t_1 + v_{s_3}\Delta t - \Delta x}{v_{s_3}-v_{s_2}}\right) &= (\lambda_{s_1}-\lambda_{s_2})(\lambda_{s_3}-\lambda_{s_1})(\lambda_{s_3}-\lambda_{s_2})\exp\left(\Delta t(\lambda_{s_1}+\lambda_{s_2})\right) \\ &\quad\cdot \exp\left(t_1(\lambda_{s_3}-\lambda_{s_1}) + \frac{(v_{s_1}-v_{s_3})t_1 + v_{s_3}\Delta t - \Delta x}{v_{s_3}-v_{s_2}}(\lambda_{s_3}-\lambda_{s_2})\right) \\ &= k_{1,2,3}\cdot \tilde{M}(\Delta x)\cdot \tilde{N}(t_1),\end{aligned}$$

which was written denoting

$$k_{1,2,3} := -(\lambda_{s_1} - \lambda_{s_2})(\lambda_{s_2} - \lambda_{s_3})(\lambda_{s_3} - \lambda_{s_1}) \cdot \exp\left(\frac{v_{s_2}(\lambda_{s_1} + \lambda_{s_2}) - v_{s_3}(\lambda_{s_1} + \lambda_{s_3})}{v_{s_2} - v_{s_3}}\Delta t\right),$$

$$\tilde{M}(\Delta x) := \exp\left(\frac{-(\lambda_{s_3} - \lambda_{s_2})}{v_{s_3} - v_{s_2}}\Delta x\right),$$

and

$$\begin{aligned}\tilde{N}(t_1) :=& \exp\left(\left(\lambda_{s_3} - \lambda_{s_1} + \frac{(v_{s_1} - v_{s_3})}{v_{s_3} - v_{s_2}}(\lambda_{s_3} - \lambda_{s_2})\right) t_1\right)\\ =& \exp\left(\frac{v_{s_1}(\lambda_{s_3} - \lambda_{s_2}) + v_{s_2}(\lambda_{s_1} - \lambda_{s_3}) + v_{s_3}(\lambda_{s_2} - \lambda_{s_1})}{v_{s_3} - v_{s_2}} t_1\right)\\ =& \exp\left(\frac{\nu_{1,2,3}}{v_{s_2} - v_{s_3}} t_1\right),\end{aligned}$$

where

$$\nu_{1,2,3} := v_{s_1}(\lambda_{s_2} - \lambda_{s_3}) + v_{s_2}(\lambda_{s_3} - \lambda_{s_1}) + v_{s_3}(\lambda_{s_1} - \lambda_{s_2}).$$

Now, we define and compute the indefinite integral in t_1

$$\begin{aligned}I(t_1) :=& \int_{t_1} \frac{1}{|v_{s_3} - v_{s_2}|}\, \bar{g}_{s_1,s_2,s_3}\left(t_1, \frac{(v_{s_1} - v_{s_3})t_1 + v_{s_3}\Delta t - \Delta x}{v_{s_3} - v_{s_2}}\right) \mathrm{d}t_1\\ =& \frac{1}{|v_{s_3} - v_{s_2}|}\frac{k_{1,2,3}}{D}\tilde{M}(\Delta x)\int_{t_1}\tilde{N}(t_1)\mathrm{d}t_1\\ =& \frac{k_{1,2,3}\tilde{M}(\Delta x)}{|v_{s_3} - v_{s_2}| \cdot D}\frac{v_{s_2} - v_{s_3}}{\nu_{1,2,3}}\tilde{N}(t_1)\\ =& \frac{k_{1,2,3}\tilde{M}(\Delta x)}{D}\frac{\mathrm{sign}(v_{s_2} - v_{s_3})}{\nu_{1,2,3}}\tilde{N}(t_1),\end{aligned}$$

in which the result of the indefinite integral is obtained up to a constant.

We now need to determine area of integration, which depend on Δx and on the increasing order assumed by the three velocities. Hence, we first denote $v_{\min}, v_{\text{int}}, v_{\max} \in \{v_{s_1}, v_{s_2}, v_{s_3}\}$ such that $v_{\min} < v_{\text{int}} < v_{\max}$. Now, the probability density of Δx is non-zero for $\Delta x \in (v_{\min}\Delta t, v_{\max}\Delta t)$.

We denote the domain of integration for t_1

$$A := \left\{t_1 \;\middle|\; t_1 \in [0, \Delta t], \frac{(v_{s_1} - v_{s_3})t_1 + v_{s_3}\Delta t - \Delta x}{v_{s_3} - v_{s_2}} \in [0, \Delta t - t_1]\right\},$$

and we note that if A is not empty, it is an interval $A = [E_0, E_1]$. Since $t_1 \in [0, \Delta t]$ then $E_0 \geq 0$ and $E_1 \leq \Delta t$. Moreover,

$$t_2 = \frac{\Delta x - (v_{s_1} - v_{s_3})t_1 - v_{s_3}\Delta t}{v_{s_2} - v_{s_3}} \in [0, \Delta t - t_1].$$

gives other conditions on the area of integration:

$$\begin{cases}(v_{s_1} - v_{s_3})t_1 + v_{s_3}\Delta t \leq \Delta x \leq (v_{s_1} - v_{s_2})t_1 + v_{s_2}\Delta t, & \text{if } v_{s_2} > v_{s_3},\\ (v_{s_1} - v_{s_2})t_1 + v_{s_2}\Delta t \leq \Delta x \leq (v_{s_1} - v_{s_3})t_1 + v_{s_3}\Delta t, & \text{if } v_{s_2} < v_{s_3}.\end{cases} \tag{S3}$$

We define

$$a_1 := \frac{\Delta x - v_{s_2}\Delta t}{v_{s_1} - v_{s_2}},$$

and

$$b_1 := \frac{\Delta x - v_{s_3}\Delta t}{v_{s_1} - v_{s_3}},$$

and we notice that we can write the conditions in Equation (S3) as

$$\begin{cases} \text{if } v_{s_2} > v_{s_3}, & \begin{cases} t_1 \le b_1 & \text{if } v_{s_1} > v_{s_3}, \\ t_1 \ge b_1 & \text{if } v_{s_1} < v_{s_3}, \end{cases} \text{ and } \begin{cases} t_1 \ge a_1 & \text{if } v_{s_1} > v_{s_2}, \\ t_1 \le a_1 & \text{if } v_{s_1} < v_{s_2}, \end{cases} \\ \text{if } v_{s_2} < v_{s_3}, & \begin{cases} t_1 \ge b_1 & \text{if } v_{s_1} > v_{s_3}, \\ t_1 \le b_1 & \text{if } v_{s_1} < v_{s_3}, \end{cases} \text{ and } \begin{cases} t_1 \le a_1 & \text{if } v_{s_1} > v_{s_2}, \\ t_1 \ge a_1 & \text{if } v_{s_1} < v_{s_2}. \end{cases} \end{cases}$$

We apply the lower boundaries for t_1 to determine E_0 and upper boundaries to determine E_1, which depend on whether $\Delta x \in [v_{\min}\Delta t, v_{\text{int}}\Delta t]$ or $\Delta x \in [v_{\text{int}}\Delta t, v_{\max}\Delta t]$. In particular, we obtain

$$\begin{cases} \text{if } v_{s_2} > v_{s_1} > v_{s_3}, & \begin{cases} E_0 = 0,\ E_1 = b_1, & \text{if } \Delta x \le v_{\text{int}}\Delta t, \\ E_0 = 0,\ E_1 = a_1, & \text{if } \Delta x \ge v_{\text{int}}\Delta t, \end{cases} \\ \text{if } v_{s_3} > v_{s_1} > v_{s_2}, & \begin{cases} E_0 = 0,\ E_1 = a_1, & \text{if } \Delta x \le v_{\text{int}}\Delta t, \\ E_0 = 0,\ E_1 = b_1, & \text{if } \Delta x \ge v_{\text{int}}\Delta t, \end{cases} \\ \text{if } v_{s_1} > v_{s_2} > v_{s_3}, & \begin{cases} E_0 = 0,\ E_1 = a_1, & \text{if } \Delta x \le v_{\text{int}}\Delta t, \\ E_0 = b_1,\ E_1 = a_1, & \text{if } \Delta x \ge v_{\text{int}}\Delta t, \end{cases} \\ \text{if } v_{s_3} > v_{s_2} > v_{s_1}, & \begin{cases} E_0 = a_1,\ E_1 = b_1, & \text{if } \Delta x \le v_{\text{int}}\Delta t, \\ E_0 = 0,\ E_1 = b_1, & \text{if } \Delta x \ge v_{\text{int}}\Delta t, \end{cases} \\ \text{if } v_{s_1} > v_{s_3} > v_{s_2}, & \begin{cases} E_0 = 0,\ E_1 = a_1, & \text{if } \Delta x \le v_{\text{int}}\Delta t, \\ E_0 = b_1,\ E_1 = a_1, & \text{if } \Delta x \ge v_{\text{int}}\Delta t, \end{cases} \\ \text{if } v_{s_2} > v_{s_3} > v_{s_1}, & \begin{cases} E_0 = b_1,\ E_1 = a_1, & \text{if } \Delta x \le v_{\text{int}}\Delta t, \\ E_0 = 0,\ E_1 = a_1, & \text{if } \Delta x \ge v_{\text{int}}\Delta t. \end{cases} \end{cases}$$

In conclusion,

$$\tilde{g}_{s_1,s_2,s_3}(\Delta x) := \mathbb{P}(\Delta x \,|\, W = 2, s_1, s_2, s_3) = I(E_1) - I(E_0).$$

S5.3 Computing $\mathbb{P}(\Delta y \,|\, W = 2, S_1 = s_1, S_2 = s_2, S_3 = s_3),\ s_3 \neq s_1$

By writing $\tilde{g}$ explicitly, it can be verified that

$$\tilde{g}_{s_1,s_2,s_3}(\Delta x) = \tilde{g}_{s^{(i_1)},s^{(i_2)},s^{(i_3)}}(\Delta x),$$

for any $i_1, i_2, i_3 \in \{1, 2, 3\}$ all distinct. In other words, once three distinct states (with distinct rates and velocities) are fixed, the probability distribution of obtaining a Δx with two switches does not depend on the order of the states are attained. Hence, we can compute

$$\begin{aligned} \tilde{f}_{s_1,s_2,s_3}(\Delta y) &:= \mathbb{P}(\Delta y \,|\, W = 2, S_1 = s_1, S_2 = s_2, S_3 = s_3) \\ &= \int_{v_{\min}\Delta t}^{v_{\max}\Delta t} \tilde{g}_{s_1,s_2,s_3}(\Delta x) f_{\mathcal{N}(0,2\sigma^2)}(\Delta y - \Delta x) \mathrm{d}(\Delta x), \end{aligned}$$

by considering the simplest form for

$$\tilde{g}_{s_1,s_2,s_3}(\Delta x) = I_{s_1,s_2,s_3}(E_1) - I_{s_1,s_2,s_3}(0),$$

where we fix $v_{s_2} = v_{\max}$, $v_{s_1} = v_{\text{int}}$, $v_{s_3} = v_{\min}$, and rearrange the rates λ_{s_i} accordingly, and

$$\begin{cases} E_1 = b_1 = \dfrac{\Delta x - v_{s_3}\Delta t}{v_{s_1} - v_{s_3}}, & \text{if } \Delta x \leq v_{\text{int}}\Delta t, \\ E_1 = a_1 = \dfrac{\Delta x - v_{s_2}\Delta t}{v_{s_1} - v_{s_2}}, & \text{if } \Delta x \geq v_{\text{int}}\Delta t. \end{cases}$$

Therefore,

$$\begin{aligned} \tilde{f}_{s_1,s_2,s_3}(\Delta y) = & \int_{v_{\min}\Delta t}^{v_{\text{int}}\Delta t} \tilde{g}_{s_1,s_2,s_3}(\Delta x) f_{\mathcal{N}(0,2\sigma^2)}(\Delta y - \Delta x)\mathrm{d}(\Delta x) \\ & + \int_{v_{\text{int}}\Delta t}^{v_{\max}\Delta t} \tilde{g}_{s_1,s_2,s_3}(\Delta x) f_{\mathcal{N}(0,2\sigma^2)}(\Delta y - \Delta x)\mathrm{d}(\Delta x) \\ = & \int_{v_{\min}\Delta t}^{v_{\text{int}}\Delta t} (I(b_1) - I(0)) f_{\mathcal{N}(0,2\sigma^2)}(\Delta y - \Delta x)\mathrm{d}(\Delta x) \\ & + \int_{v_{\text{int}}\Delta t}^{v_{\max}\Delta t} (I(a_1) - I(0)) f_{\mathcal{N}(0,2\sigma^2)}(\Delta y - \Delta x)\mathrm{d}(\Delta x) \\ = & \int_{v_{\min}\Delta t}^{v_{\max}\Delta t} -I(0) f_{\mathcal{N}(0,2\sigma^2)}(\Delta y - \Delta x)\mathrm{d}(\Delta x), \\ & + \int_{v_{\min}\Delta t}^{v_{\text{int}}\Delta t} I(b_1) f_{\mathcal{N}(0,2\sigma^2)}(\Delta y - \Delta x)\mathrm{d}(\Delta x) \\ & + \int_{v_{\text{int}}\Delta t}^{v_{\max}\Delta t} I(a_1) f_{\mathcal{N}(0,2\sigma^2)}(\Delta y - \Delta x)\mathrm{d}(\Delta x) \\ =: & \, J_0 + J_{b_1} + J_{a_1}, \end{aligned}$$

where

$$f_{\mathcal{N}(0,2\sigma^2)}(\Delta\epsilon) := \frac{1}{2\sigma\sqrt{\pi}} \exp\left(-\frac{(\Delta\epsilon)^2}{4\sigma^2}\right),$$

and

$$\begin{aligned} I(t_1) &= \frac{k_{1,2,3}\tilde{M}(\Delta x)}{D} \frac{\operatorname{sign}(v_{s_2} - v_{s_3})}{\nu_{1,2,3}} \tilde{N}(t_1), \\ \tilde{N}(t_1) &= \exp\left(\frac{\nu_{1,2,3}}{v_{s_2} - v_{s_3}} t_1\right), \\ \tilde{M}(\Delta x) &= \exp\left(\frac{\lambda_{s_2} - \lambda_{s_3}}{v_{s_3} - v_{s_2}} \Delta x\right). \end{aligned}$$

By defining

$$\tilde{k} := \frac{k_{1,2,3}}{D} \frac{\operatorname{sign}(v_{s_2} - v_{s_3})}{\nu_{1,2,3}} \frac{1}{2\sigma\sqrt{\pi}},$$

then we write

$$\begin{aligned} J_0 := & \int_{v_{\min}\Delta t}^{v_{\max}\Delta t} -I(0) f_{\mathcal{N}(0,2\sigma^2)}(\Delta y - \Delta x)\mathrm{d}(\Delta x) \\ = & \int_{v_{\min}\Delta t}^{v_{\max}\Delta t} -\tilde{k} \exp\left(\frac{\lambda_{s_2} - \lambda_{s_3}}{v_{s_3} - v_{s_2}} \Delta x\right) \exp\left(-\frac{(\Delta y - \Delta x)^2}{4\sigma^2}\right) \mathrm{d}(\Delta x) \\ = & \, k_0 \left[\operatorname{erf}\left(\frac{2c(\Delta x) - r_0}{2\sqrt{c}}\right)\right]_{\Delta x = v_{\min}\Delta t}^{\Delta x = v_{\max}\Delta t}, \end{aligned}$$

where

$$\begin{aligned} c &:= \frac{1}{4\sigma^2}, \\ r_0 = r_0(\Delta y) &:= 2c\Delta y + \frac{\lambda_{s_2} - \lambda_{s_3}}{v_{s_3} - v_{s_2}}, \end{aligned}$$

$$\hat{r}_0 = \hat{r}_0(\Delta y) := -c(\Delta y)^2,$$

and

$$\begin{aligned} k_0 :=& -\tilde{k}\frac{\sqrt{\pi}}{2\sqrt{c}} \exp\left(\frac{r_0^2}{4c} + \hat{r}_0\right) \\ =& -\frac{k_{1,2,3}}{D}\frac{\operatorname{sign}(v_{s_2} - v_{s_3})}{\nu_{1,2,3}}\frac{1}{2}\exp\left(\frac{r_0^2}{4c} + \hat{r}_0\right) \\ =& \frac{k_{1,2,3}}{D}\frac{\operatorname{sign}(v_{s_3} - v_{s_2})}{\nu_{1,2,3}}\frac{1}{2}\exp\left(\frac{r_0^2}{4c} + \hat{r}_0\right). \end{aligned}$$

Similarly,

$$\begin{aligned} J_{b_1} :=& \int_{v_{\min}\Delta t}^{v_{\text{int}}\Delta t} I(b_1) f_{\mathcal{N}(0,2\sigma^2)}(\Delta y - \Delta x)\mathrm{d}(\Delta x) \\ =& \int_{v_{\min}\Delta t}^{v_{\text{int}}\Delta t} \tilde{k}\tilde{N}\left(\frac{\Delta x - v_{s_3}\Delta t}{v_{s_1} - v_{s_3}}\right)\exp\left(\frac{-(\lambda_{s_3} - \lambda_{s_2})}{v_{s_3} - v_{s_2}}\Delta x\right)\exp\left(-\frac{(\Delta y - \Delta x)^2}{4\sigma^2}\right)\mathrm{d}(\Delta x) \\ =& k_{b_1}\left[\operatorname{erf}\left(\frac{2c(\Delta x) - r_{b_1}}{2\sqrt{c}}\right)\right]_{\Delta x = v_{\min}\Delta t}^{\Delta x = v_{\text{int}}\Delta t}, \end{aligned}$$

but now

$$\begin{aligned} r_{b_1} = r_{b_1}(\Delta y) :=& 2c\Delta y + \frac{\lambda_{s_2} - \lambda_{s_3}}{v_{s_3} - v_{s_2}} + \frac{\nu_{1,2,3}}{(v_{s_2} - v_{s_3})(v_{s_1} - v_{s_3})} \\ =& r_0 + \frac{\nu_{1,2,3}}{(v_{s_2} - v_{s_3})(v_{s_1} - v_{s_3})}, \\ \hat{r}_{b_1} :=& -c(\Delta y)^2 - \frac{\nu_{1,2,3}\cdot v_{s_3}\Delta t}{(v_{s_2} - v_{s_3})(v_{s_3} - v_{s_1})} \\ =& \hat{r}_0 - \frac{\nu_{1,2,3}\cdot v_{s_3}\Delta t}{(v_{s_3} - v_{s_2})(v_{s_3} - v_{s_1})}, \end{aligned}$$

and

$$\begin{aligned} k_{b_1} :=& \tilde{k}\frac{\sqrt{\pi}}{2\sqrt{c}}\exp\left(\frac{r_{b_1}^2}{4c} + \hat{r}_{b_1}\right) \\ =& \frac{k_{1,2,3}}{D}\frac{\operatorname{sign}(v_{s_2} - v_{s_3})}{\nu_{1,2,3}}\frac{1}{2}\exp\left(\frac{r_{b_1}^2}{4c} + \hat{r}_{b_1}\right). \end{aligned}$$

Finally,

$$\begin{aligned} J_{a_1} :=& \int_{v_{\text{int}}\Delta t}^{v_{\max}\Delta t} I(a_1) f_{\mathcal{N}(0,2\sigma^2)}(\Delta y - \Delta x)\mathrm{d}(\Delta x) \\ =& \int_{v_{\text{int}}\Delta t}^{v_{\max}\Delta t} \tilde{k}\tilde{N}\left(\frac{\Delta x - v_{s_2}\Delta t}{v_{s_1} - v_{s_2}}\right)\exp\left(\frac{-(\lambda_{s_3} - \lambda_{s_2})}{v_{s_3} - v_{s_2}}\Delta x\right)\exp\left(-\frac{(\Delta y - \Delta x)^2}{4\sigma^2}\right)\mathrm{d}(\Delta x) \\ =& k_{a_1}\left[\operatorname{erf}\left(\frac{2c(\Delta x) - r_{a_1}}{2\sqrt{c}}\right)\right]_{\Delta x = v_{\text{int}}\Delta t}^{\Delta x = v_{\max}\Delta t}, \end{aligned}$$

but now

$$\begin{aligned} r_{a_1} = r_{a_1}(\Delta y) :=& 2c\Delta y + \frac{\lambda_{s_2} - \lambda_{s_3}}{v_{s_3} - v_{s_2}} + \frac{\nu_{1,2,3}}{(v_{s_2} - v_{s_3})(v_{s_1} - v_{s_2})} \\ =& r_0 + \frac{\nu_{1,2,3}}{(v_{s_2} - v_{s_3})(v_{s_1} - v_{s_2})}, \\ \hat{r}_{a_1} = \hat{r}_{a_1}(\Delta y) :=& -c(\Delta y)^2 - \frac{\nu_{1,2,3}\cdot v_{s_2}\Delta t}{(v_{s_2} - v_{s_3})(v_{s_1} - v_{s_2})} \\ =& \hat{r}_0 - \frac{\nu_{1,2,3}\cdot v_{s_2}\Delta t}{(v_{s_2} - v_{s_3})(v_{s_1} - v_{s_2})}, \end{aligned}$$

and

$$k_{a_1} := \tilde{k}\frac{\sqrt{\pi}}{2\sqrt{c}}\exp\left(\frac{r_{a_1}^2}{4c}+\hat{r}_{a_1}\right)$$
$$= \frac{k_{1,2,3}}{D}\frac{\operatorname{sign}(v_{s_2}-v_{s_3})}{\nu_{1,2,3}}\frac{1}{2}\exp\left(\frac{r_{a_1}^2}{4c}+\hat{r}_{a_1}\right).$$

S5.4 Computing $\mathbb{P}(W = 2 \,|\, S_1 = s_1, S_2 = s_2, S_3 = s_3)$ for $s_3 = s_1$

Here, we compute $\mathbb{P}(W = 2 \,|\, S_1 = s_1, S_2 = s_2, S_3 = s_1)$. Since $S_3 = S_1$, we only need to define a function of $t_2 \in (0, \Delta t]$

$$\begin{aligned}
F_{s_1,s_2,s_1}(t_2) &:= \mathbb{P}(\tau_2 \le t_2, 0 < \tau_1 \le \Delta t, 0 < \tau_2 \le \Delta t - \tau_1, \tau_3 > \Delta t - \tau_1 - \tau_2 \,|\, S_1 = s_1, S_2 = s_2, S_3 = s_1)\\
&= \mathbb{P}(0 \le \tau_2 \le t_2, 0 < \tau_1 \le \Delta t - \tau_2, \tau_3 > \Delta t - \tau_1 - \tau_2 \,|\, S_1 = s_1, S_2 = s_2, S_3 = s_1)\\
&= \int_0^{t_2}\left(\int_0^{\Delta t-\tau_2}\left(\int_{\Delta t-\tau_1-\tau_2}^{\infty} f_{s_1,s_2,s_1}(\tau_1,\tau_2,\tau_3)\mathrm{d}\tau_3\right)\mathrm{d}\tau_1\right)\mathrm{d}\tau_2\\
&= \frac{\lambda_{s_1}\lambda_{s_2}\exp(-\lambda_{s_1}\Delta t)}{(\lambda_{s_1}-\lambda_{s_2})^2}\Big(((\lambda_{s_1}-\lambda_{s_2})\Delta t(\exp((\lambda_{s_1}-\lambda_{s_2})t_2)-1))\\
&\qquad - ((\lambda_{s_1}-\lambda_{s_2})t_2 - 1)\exp((\lambda_{s_1}-\lambda_{s_2})t_2) - 1\Big),
\end{aligned}$$

in the assumption $\lambda_{s_1} \neq \lambda_{s_2}$. Therefore, in the previous notation we obtain

$$\begin{aligned}
G_{s_1,s_2,s_1}(t_2) &:= \mathbb{P}(\tau_2 \le t_2 \,|\, 0 < \tau_1 \le \Delta t, 0 < \tau_2 \le \Delta t - \tau_1, \tau_3 > \Delta t - \tau_1 - \tau_2, S_1 = s_1, S_2 = s_2, S_3 = s_1)\\
&= \frac{F_{s_1,s_2,s_1}(t_2)}{F_{s_1,s_2,s_1}(\Delta t)}\\
&= \frac{(\lambda_{s_1}-\lambda_{s_2})\Delta t(\exp((\lambda_{s_1}-\lambda_{s_2})t_2)-1) - ((\lambda_{s_1}-\lambda_{s_2})t_2-1)\exp((\lambda_{s_1}-\lambda_{s_2})t_2) - 1}{\exp((\lambda_{s_1}-\lambda_{s_2})\Delta t) - 1 - (\lambda_{s_1}-\lambda_{s_2})\Delta t}.
\end{aligned}$$

Finally,

$$\begin{aligned}
\mathbb{P}(W = 1 \,|\, S_1 = s_1, S_2 = s_2, S_3 = s_1) &= F_{s_1,s_2,s_1}(\Delta t)\\
&= \frac{\lambda_{s_1}\lambda_{s_2}\exp(-\lambda_{s_1}\Delta t)}{(\lambda_{s_1}-\lambda_{s_2})^2}(\exp((\lambda_{s_1}-\lambda_{s_2})\Delta t) - 1 - (\lambda_{s_1}-\lambda_{s_2})\Delta t).
\end{aligned}$$

S5.5 Computing $\mathbb{P}(\Delta x \,|\, W = 2, S_1 = s_1, S_2 = s_2, S_3 = s_3)$ for $s_3 = s_1$

From the CDF $G_{s_1,s_2,s_1}(t_2)$ we can obtain the PDF

$$\begin{aligned}
g_{s_1,s_2,s_1}(t_2) := \mathbb{P}(t_2 \,|\, W = 2, S_1 = s_1, S_2 = s_2, S_3 = s_1) &= \frac{\mathrm{d}}{\mathrm{d}t_2}G_{s_1,s_2,s_1}(t_2)\\
&= \frac{(\lambda_{s_1}-\lambda_{s_2})^2(\Delta t - t_2)\exp((\lambda_{s_1}-\lambda_{s_2})t_2)}{\exp((\lambda_{s_1}-\lambda_{s_2})\Delta t) - 1 - (\lambda_{s_1}-\lambda_{s_2})\Delta t}.
\end{aligned}$$

Now, we approach the problem similarly to Section S4.3.

We define

$$h_{s_1,s_2,s_1}(t_2) := v_{s_2}t_2 + v_{s_1}(\Delta t - t_2).$$

We note that $h_{s_1,s_2,s_1} : \mathbb{R} \to \mathbb{R}$ is a monotonic function in t_2 and $\Delta x = h_{s_1,s_2,s_1}(t_2)$. Thus the PDF of Δx is

$$\begin{aligned}
\tilde{g}_{s_1,s_2,s_1}(\Delta x) &= \mathbb{P}(\Delta x \,|\, W = 2, S_1 = s_1, S_2 = s_2, S_3 = s_1)\\
&= g_{s_1,s_2,s_1}\left(h^{-1}_{s_1,s_2,s_1}(\Delta x)\right)\left|\frac{\mathrm{d}}{\mathrm{d}(\Delta x)}h^{-1}_{s_1,s_2,s_1}(\Delta x)\right|,
\end{aligned}$$

where

$$h^{-1}_{s_1,s_2,s_1}(\Delta x) = \frac{\Delta x - v_{s_1}\Delta t}{v_{s_2} - v_{s_1}},$$

and its derivative is

$$\frac{\mathrm{d}}{\mathrm{d}(\Delta x)} h^{-1}_{s_1,s_2,s_1}(\Delta x) = \frac{1}{v_{s_2} - v_{s_1}}.$$

Hence,

$$\tilde{g}_{s_1,s_2,s_1}(\Delta x) = \frac{(\lambda_{s_1} - \lambda_{s_2})^2}{|v_{s_2} - v_{s_1}|} \cdot \frac{-(\Delta x - v_{s_2}\Delta t)}{v_{s_2} - v_{s_1}} \cdot \frac{\exp\left((\lambda_{s_1} - \lambda_{s_2})(\Delta x - v_{s_1}\Delta t)/(v_{s_2} - v_{s_1})\right)}{\exp\left((\lambda_{s_1} - \lambda_{s_2})\Delta t\right) - 1 - (\lambda_{s_1} - \lambda_{s_2})\Delta t}.$$

S5.6 Computing $\mathbb{P}(\Delta y \,|\, W = 2, S_1 = s_1, S_2 = s_2, S_3 = s_3)$ for $s_3 = s_1$

By convoluting with the PDF for $\Delta\epsilon$ we now incorporate noise

$$\begin{aligned}
\tilde{f}_{s_1,s_2,s_1}(\Delta y) &:= \mathbb{P}(\Delta y \,|\, W = 1, S_1 = s_1, S_2 = s_2, S_3 = s_1) \\
&= \int_a^b \tilde{g}_{s_1,s_2,s_1}(\Delta x) f_{\mathcal{N}(0,2\sigma^2)}(\Delta y - \Delta x)\mathrm{d}(\Delta x),
\end{aligned}$$

where

$$\begin{aligned}
a = a_{s_1,s_2,s_1} &:= \min\{h_{s_1,s_2,s_1}(0), h_{s_1,s_2,s_1}(\Delta t)\}, \\
b = b_{s_1,s_2,s_1} &:= \max\{h_{s_1,s_2,s_1}(0), h_{s_1,s_2,s_1}(\Delta t)\},
\end{aligned}$$

and

$$f_{\mathcal{N}(0,2\sigma^2)}(\Delta\epsilon) := \frac{1}{2\sigma\sqrt{\pi}} \exp\left(-\frac{(\Delta\epsilon)^2}{4\sigma^2}\right).$$

We obtain

$$\begin{aligned}
\tilde{f}_{s_1,s_2,s_1}(\Delta y) &= \hat{k} \int_a^b (-v_{s_2}\Delta t + \Delta x) \exp\left(-c(\Delta x)^2 + r\Delta x + \hat{r}\right)\mathrm{d}(\Delta x) \\
&= \frac{-\hat{k}e^g}{4c^{3/2}} \left[\sqrt{\pi}e^{\frac{r^2}{4c}}(2c(-v_{s_2}\Delta t) + r)\mathrm{erf}\left(\frac{2c\Delta x - r}{2\sqrt{c}}\right) - 2\sqrt{c}e^{\Delta x(r - c\Delta x)}\right]_{\Delta x = a}^{\Delta x = b} \\
&= k \left[\sqrt{\pi}e^{\frac{r^2}{4c}}(2c(-v_{s_2}\Delta t) + r)\mathrm{erf}\left(\frac{2c\Delta x - r}{2\sqrt{c}}\right) - 2\sqrt{c}e^{\Delta x(r - c\Delta x)}\right]_{\Delta x = a}^{\Delta x = b},
\end{aligned}$$

where

$$\begin{aligned}
c &:= \frac{1}{4\sigma^2}, \\
r = r_{s_1,s_2,s_1}(\Delta y) &:= 2c\Delta y + \frac{\lambda_{s_1} - \lambda_{s_2}}{v_{s_2} - v_{s_1}}, \\
\hat{r} = \hat{r}_{s_1,s_2,s_1}(\Delta y) &:= -c(\Delta y)^2 + \frac{\lambda_{s_1} - \lambda_{s_2}}{|v_{s_2} - v_{s_1}|}|v_{s_1}|\Delta t, \\
\hat{k} = \hat{k}_{s_1,s_2,s_1} &:= \frac{(\lambda_{s_1} - \lambda_{s_2})^2}{2\sigma\sqrt{\pi}|v_{s_2} - v_{s_1}|^2} \frac{\mathrm{sign}(v_{s_2} - v_{s_1})}{\exp\left((\lambda_{s_1} - \lambda_{s_2})\Delta t\right) - 1 - (\lambda_{s_1} - \lambda_{s_2})\Delta t},
\end{aligned}$$

and

$$\begin{aligned}
k = k_{s_1,s_2,s_1} &:= \frac{-\hat{k}e^{\hat{r}}}{4c^{3/2}} \\
&= \frac{\sigma^2 e^{\hat{r}}(\lambda_{s_1} - \lambda_{s_2})^2}{\sqrt{\pi}|v_{s_2} - v_{s_1}|^2} \frac{-\mathrm{sign}(v_{s_2} - v_{s_1})}{\exp\left((\lambda_{s_1} - \lambda_{s_2})\Delta t\right) - 1 - (\lambda_{s_1} - \lambda_{s_2})\Delta t}.
\end{aligned}$$

S5.7 Comparison of the error of the up-to-one-switch approximation with the error of the up-to-two-switch approximation

Figure S3 shows a comparison of the up-to-one-switch approximation error, $P_1(\Delta y) - P(\Delta y)$, with the up-to-two-switch approximation error, $P_1(\Delta y) - P(\Delta y)$, for the two-state model and the three-state model.

Figure S4 shows a comparison between the empirical distributions for $\mathbb{P}(\Delta y|W = 2)$ and $\mathbb{P}(\Delta y|W > 2)$, using the rates $10\boldsymbol{\lambda}$ for $\boldsymbol{\lambda}$ specified in Supplementary Information Figure S1, for the two-state model (from Figure 2**A**) and three-state model (from Figure 2**B**). Comparing the two panel suggests that the approximation used ($\mathbb{P}(\Delta y \,|\, W > 2) \approx \mathbb{P}(\Delta y \,|\, W = 2)$) is less accurate for the two-state model in this parameter regime, compared to the three-state model. And this causes the up-to-two-switch approximation to be more accurate for the three-state model (panel **F** in Figure 7) than for the two-state model in (panel **E** in Figure 7) for frequent switching.

S6 Up-to-one-switch approximation for the probability distribution function of a set of noisy subsequent location increments

In this section, we extend the results obtained in Section 3.1 to compute an approximation for the PDF a set of N noisy subsequent location increments $\mathbb{P}(\boldsymbol{\Delta y}_N)$. Here, we compute explicitly all the results obtained in Section 3.2. The up-to-two-switch approximation of a single location increment is provided as a Python code in the function *approx_pdf_track_up_to_1_switch* in the file *functions.py*.

The PDF $\mathbb{P}(\boldsymbol{\Delta y}_N)$ is the joint distribution of a set of subsequent location increments, and it can be rewritten in terms of conditional distributions

$$\mathbb{P}(\boldsymbol{\Delta y}_N) = \mathbb{P}(\Delta y_1)\mathbb{P}(\Delta y_2 \,|\, \boldsymbol{\Delta y}_1) \cdots \mathbb{P}(\Delta y_{N-1} \,|\, \boldsymbol{\Delta y}_{N-2})\mathbb{P}(\Delta y_N \,|\, \boldsymbol{\Delta y}_{N-1}),$$

where we note that, for an increment Δy, the PDF $\mathbb{P}(\Delta y)$ is its marginal distribution, approximated in Section 3.1 and Section 3.2. We obtain that for any $N \geq 2$ we can approximate the joint distribution of a set of subsequent location increments using a recursive method. We denote S_i^j the i-th state attained and W^j the number of switches the during the j-th measured interval of the track (as in Figure 8).

We compute an iterative formula for $\mathbb{P}(\Delta y_N \,|\, \boldsymbol{\Delta y}_{N-1})$ that involves the approximations for

$$\mathbb{P}(\Delta y_N \,|\, S_1^N = s_1^N),\ \mathbb{P}(W^N = 0 \,|\, S_1^N = s_1^N),\ \mathbb{P}(W^N \geq 1 \,|\, S_1^N = s_1^N),\ \mathbb{P}(S_1^N = s_1^N \,|\, \boldsymbol{\Delta y}_{N-1}),$$

using $\mathbb{P}(\Delta y_{N-1} \,|\, \boldsymbol{\Delta y}_{N-2})$ and

$$\begin{aligned}&\mathbb{P}(\Delta y_{N-1} \,|\, S_1^{N-1} = s_1^{N-1}),\ \mathbb{P}(W^{N-1} = 0 \,|\, S_1^{N-1}),\\ &\mathbb{P}(W^{N-1} \geq 1 \,|\, S_1^{N-1} = s_1^{N-1}),\ \mathbb{P}(S_1^{N-1} = s_1^{N-1} \,|\, \boldsymbol{\Delta y}_{N-2}).\end{aligned}$$

We note that, for $N = 2$, $\mathbb{P}(\Delta y_{N-1} \,|\, \boldsymbol{\Delta y}_{N-2})$ is the marginal PDF, for which we have computed approximations. For simplicity, we use the up-to-one-switch approximation presented in Section 3.1 which we denote by P_1, however, the result could be extended to incorporate more switches.

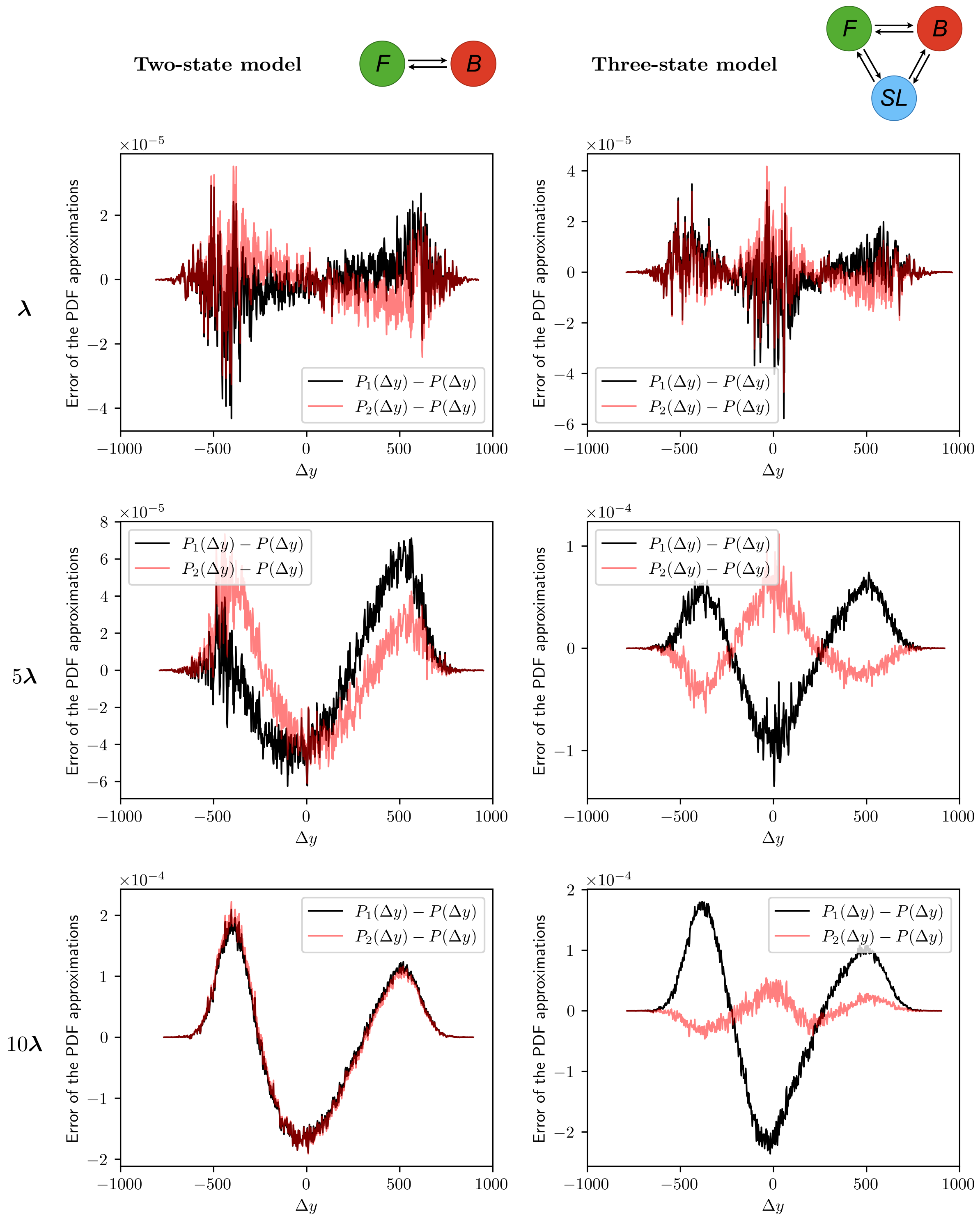

Fig. S3 The error of the approximation $P_1(\Delta y)$, defined as $P_1(\Delta y) - P(\Delta y)$ where $P(\Delta y)$ is the empirical PDF for Δy, is compared with the error of the approximation $P_1(\Delta y)$, defined as $P_1(\Delta y) - P(\Delta y)$. The panels at the top are obtained using the parameters as specified in the Supplementary Information Figure S1, while the panels in the middle and at the bottom are obtained with the same parameters except the rates which are multiplied by 5 and 10, respectively.

Using the state at the beginning of the N-th interval S_1^N, we write

$$\mathbb{P}(\Delta y_N \,|\, \boldsymbol{\Delta y}_{N-1}) = \sum_{s_1^N=1}^{n} \mathbb{P}(\Delta y_N \,|\, S_1^N = s_1^N, \boldsymbol{\Delta y}_{N-1}) \mathbb{P}(S_1^N = s_1^N \,|\, \boldsymbol{\Delta y}_{N-1}).$$

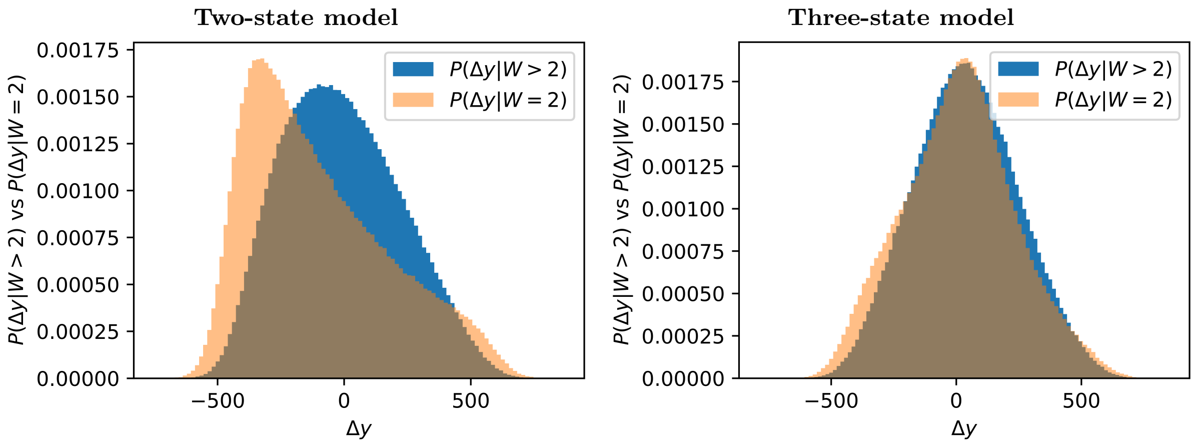

Fig. S4 Comparison of the empirical distributions for $\mathbb{P}(\Delta y|W=2)$ and $\mathbb{P}(\Delta y|W>2)$, using the rates $10\boldsymbol{\lambda}$ for $\boldsymbol{\lambda}$ specified in Supplementary Information Figure S1, for the two-state model (from Figure 2**A**) and three-state model (from Figure 2**B**).

In the exact increments form, we have

$$\mathbb{P}(\Delta x_N \,|\, S_1^N = s_1^N, \boldsymbol{\Delta x}_{N-1}) = \mathbb{P}(\Delta x_N \,|\, S_1^N = s_1^N),$$

which follows from the Markov property of the continuous-time process, and holds since the information on Δx_N given by the previous increments $\boldsymbol{\Delta x}_{N-1}$ is fully expressed by the final state at the end of the $(N-1)$-th interval which corresponds to the one at the beginning of the N-th interval S_1^N. In case of the noisy increments, we use the approximation

$$\mathbb{P}(\Delta y_N \,|\, S_1^N = s_1^N, \boldsymbol{\Delta y}_{N-1}) \approx \mathbb{P}(\Delta y_N \,|\, S_1^N = s_1^N), \tag{S4}$$

which is not exact since the previous increments contain additional information on the noise ϵ_{N-1}. Hence, we obtain

$$\mathbb{P}(\Delta y_N \,|\, \Delta y_{N-1}, \ldots, \Delta y_2, \Delta y_1) \approx \sum_{s_1^N=1}^{n} \mathbb{P}(\Delta y_N \,|\, S_1^N = s_1^N)\mathbb{P}(S_1^N = s_1^N \,|\, \boldsymbol{\Delta y}_{N-1}).$$

We obtain $\mathbb{P}(S_1^N = s_1^N \,|\, \boldsymbol{\Delta y}_{N-1})$ by using the state at the beginning of the previous interval S_1^{N-1},

$$\mathbb{P}(S_1^N = s_1^N \,|\, \boldsymbol{\Delta y}_{N-1}) = \sum_{s_1^{N-1}=1}^{n} \mathbb{P}(S_1^N = s_1^N \,|\, S_1^{N-1} = s_1^{N-1}, \boldsymbol{\Delta y}_{N-1})\mathbb{P}(S_1^{N-1} = s_1^{N-1} \,|\, \boldsymbol{\Delta y}_{N-1}).$$

Here, we note that the state S_1^{N-1} given $\boldsymbol{\Delta x}_{N-1}$ only depends on the the increment Δx_{N-1}; thus we obtain the property

$$\mathbb{P}(S_1^N = s_1^N \,|\, S_1^{N-1} = s_1^{N-1}, \boldsymbol{\Delta x}_{N-1}) = \mathbb{P}(S_1^N = s_1^N \,|\, S_1^{N-1} = s_1^{N-1}, \Delta x_{N-1}),$$

which again follows from the Markov property of the continuous-time process, and it holds since the state S_1^{N-1} given $\boldsymbol{\Delta x}_{N-1}$ only depends on the the increment Δx_{N-1}. For the noisy increment Δy we use the approximation (similar to Equation (S4))

$$\mathbb{P}(S_1^N = s_1^N \,|\, S_1^{N-1} = s_1^{N-1}, \boldsymbol{\Delta y}_{N-1}) \approx \mathbb{P}(S_1^N = s_1^N \,|\, S_1^{N-1} = s_1^{N-1}, \Delta y_{N-1}), \tag{S5}$$

to write

$$\begin{aligned}\mathbb{P}(S_1^N = s_1^N \mid \boldsymbol{\Delta y}_{N-1}) &\approx \sum_{s_1^{N-1}=1}^{n} \mathbb{P}(S_1^N = s_1^N \mid S_1^{N-1} = s_1^{N-1}, \Delta y_{N-1})\mathbb{P}(S_1^{N-1} \mid \boldsymbol{\Delta y}_{N-1})\\ &= \sum_{s_1^{N-1}=1}^{n} \frac{\mathbb{P}(\Delta y_{N-1}, S_1^N = s_1^N \mid S_1^{N-1} = s_1^{N-1})}{\mathbb{P}(\Delta y_{N-1} \mid S_1^{N-1} = s_1^{N-1})}\mathbb{P}(S_1^{N-1} = s_1^{N-1} \mid \boldsymbol{\Delta y}_{N-1}),\end{aligned}$$

where the equality is obtained by definition of conditional probability. The denominator $\mathbb{P}(\Delta y_{N-1} \mid S_1^{N-1} = s_1^{N-1})$ will be simplified later. Moreover, using Bayes' theorem, we write

$$\begin{aligned}\mathbb{P}(S_1^{N-1} = s_1^{N-1} \mid \boldsymbol{\Delta y}_{N-1}) &= \mathbb{P}(\Delta y_{N-1} \mid S_1^{N-1} = s_1^{N-1}, \boldsymbol{\Delta y}_{N-2})\frac{\mathbb{P}(S_1^{N-1} = s_1^{N-1} \mid \boldsymbol{\Delta y}_{N-2})}{\mathbb{P}(\Delta y_{N-1} \mid \boldsymbol{\Delta y}_{N-2})}\\ &= \mathbb{P}(\Delta y_{N-1} \mid S_1^{N-1} = s_1^{N-1})\frac{\mathbb{P}(S_1^{N-1} = s_1^{N-1} \mid \boldsymbol{\Delta y}_{N-2})}{\mathbb{P}(\Delta y_{N-1} \mid \boldsymbol{\Delta y}_{N-2})},\end{aligned}$$

where the second equality is obtained using the property in Equation (S4) (at the $(N-1)$-th step). By substitution, and, by simplifying $\mathbb{P}(\Delta y_{N-1} \mid S_1^{N-1} = s_1^{N-1})$, we obtain

$$\mathbb{P}(S_1^N = s_1^N \mid \boldsymbol{\Delta y}_{N-1}) \approx \sum_{s_1^{N-1}=1}^{n} \mathbb{P}(\Delta y_{N-1}, S_1^N = s_1^N \mid S_1^{N-1} = s_1^{N-1})\frac{\mathbb{P}(S_1^{N-1} = s_1^{N-1} \mid \boldsymbol{\Delta y}_{N-2})}{\mathbb{P}(\Delta y_{N-1} \mid \boldsymbol{\Delta y}_{N-2})}.$$

Here, the numerator and denominator of the fractions are computed at the $(N-1)$-th induction step.

Finally, we need to compute $\mathbb{P}(\Delta y_{N-1}, S_1^N = s_1^N \mid S_1^{N-1} = s_1^{N-1})$ by conditioning on the number of switches during the $(N-1)$-th interval, W^{N-1}, and we use the definition of conditional probability to obtain

$$\begin{aligned}\mathbb{P}(\Delta y_{N-1}, S_1^N = s_1^N \mid S_1^{N-1} = s_1^{N-1}) = \sum_{w=0}^{\infty} &\mathbb{P}(\Delta y_{N-1} \mid S_1^N = s_1^N, S_1^{N-1} = s_1^{N-1}, W^{N-1} = w)\\ &\times \mathbb{P}(S_1^N = s_1^N \mid S_1^{N-1} = s_1^{N-1}, W^{N-1} = w)\\ &\times \mathbb{P}(W^{N-1} = w \mid S_1^{N-1} = s_1^{N-1}).\end{aligned} \tag{S6}$$

We use the up-to-one-switch approximation and get

$$\begin{aligned}&\mathbb{P}(\Delta y_{N-1}, S_1^N = s_1^N \mid S_1^{N-1} = s_1^{N-1})\\ &\approx P_1(\Delta y_{N-1}, S_1^N = s_1^N \mid S_1^{N-1} = s_1^{N-1})\\ &= \begin{cases}\mathbb{P}(\Delta y_{N-1} \mid S_1^N = s_1^N, S_1^{N-1} = s_1^{N-1}, W^{N-1} = 0) & \\ \quad \times \mathbb{P}(W^{N-1} = 0 \mid S_1^{N-1} = s_1^{N-1}), & \text{if } s_1^N = s_1^{N-1},\\ \mathbb{P}(\Delta y_{N-1} \mid S_1^N = s_1^N, S_1^{N-1} = s_1^{N-1}, W^{N-1} = 1) & \\ \quad \times \mathbb{P}(S_2^{N-1} = s_1^N \mid S_1^{N-1} = s_1^{N-1})\mathbb{P}(W^{N-1} \geq 1 \mid S_1^{N-1} = s_1^{N-1}), & \text{if } s_1^N \neq s_1^{N-1},\end{cases}\end{aligned} \tag{S7}$$

since if $W^{N-1} = 0$, then $S_1^N = S_1^{N-1}$ which gives a unitary probability of keeping the same state

$$\mathbb{P}(S_1^N = s_1^N \mid S_1^{N-1} = s_1^{N-1}, W^{N-1} = 0) = 1,$$

while if $W^{N-1} = 1$, then we obtain $S_1^N = S_2^{N-1} \neq S_1^{N-1}$ and in the usual notation we have rewritten

$$\mathbb{P}(S_1^N = s_1^N \,|\, S_1^{N-1} = s_1^{N-1}, W^{N-1} = 1) = \mathbb{P}(S_2^{N-1} = s_1^N \,|\, S_1^{N-1} = s_1^{N-1}).$$

S6.1 Error of the up-to-one-switch approximation for the probability distribution function of two noisy subsequent location increments

Figure S5 compares the error of the up-to-one-switch approximation for the PDF of two noisy subsequent location increments, defined as $|P_1(\Delta y_1, \Delta y_2) - P(\Delta y_1, \Delta y_2)|$, with the error for the approximation consisting of the product of the marginals, defined as $|P_1(\Delta y_1)P_1(\Delta y_2) - P(\Delta y_1, \Delta y_2)|$.

S6.2 Extension to the up-to-two-switch approximation

The maximum number of switches considered in the approximation for the PDF of a set of noisy subsequent location increments can be increased by modifying Equation (S7) conditioning on a higher amount of states from Equation (S6). Using an up-to-two-switch approximation we write

$$\begin{aligned}
\mathbb{P}(\Delta y_{N-1}, S_1^N = s_1^N \,|\, S_1^{N-1} = s_1^{N-1}) &= \mathbb{P}(\Delta y_{N-1} \,|\, S_1^N = s_1^N, S_1^{N-1} = s_1^{N-1})\mathbb{P}(S_1^N = s_1^N \,|\, S_1^{N-1} = s_1^{N-1}) \\
&\approx \mathbb{P}(\Delta y_{N-1} \,|\, S_1^N = s_1^N, S_1^{N-1} = s_1^{N-1}, W^{N-1} = 0) \\
&\quad \times \mathbb{P}(S_1^N = s_1^N \,|\, S_1^{N-1} = s_1^{N-1}, W^{N-1} = 0) \\
&\quad \times \mathbb{P}(W^{N-1} = 0 \,|\, S_1^N = s_1^N, S_1^{N-1} = s_1^{N-1}) \\
&\quad + \mathbb{P}(\Delta y_{N-1} \,|\, S_1^{N-1} = s_1^N, S_1^{N-1} = s_1^{N-1}, W^{N-1} = 1) \\
&\quad \times \mathbb{P}(S_1^N = s_1^N \,|\, S_1^{N-1} = s_1^{N-1}, W^{N-1} = 1) \\
&\quad \times \mathbb{P}(W^{N-1} = 1 \,|\, S_1^N = s_1^N, S_1^{N-1} = s_1^{N-1}) \\
&\quad + \mathbb{P}(\Delta y_{N-1} \,|\, S_1^N = s_1^N, S_1^{N-1} = s_1^{N-1}, W^{N-1} = 2) \\
&\quad \times \mathbb{P}(S_1^N = s_1^N \,|\, S_1^{N-1} = s_1^{N-1}, W^{N-1} = 2) \\
&\quad \times \mathbb{P}(W^{N-1} \geq 2 \,|\, S_1^N = s_1^N, S_1^{N-1} = s_1^{N-1}).
\end{aligned}$$

Finally, this can be written as

$$\begin{aligned}
&\mathbb{P}(\Delta y_{N-1}, S_1^N = s_1^N \,|\, S_1^{N-1} = s_1^{N-1}) \\
&\approx \; \mathbb{P}(\Delta y_{N-1} \,|\, S_1^{N-1} = s_1^{N-1}, W^{N-1} = 0) \\
&\qquad \times \mathbb{P}(s_1^N = s_1^{N-1}) \\
&\qquad \times \mathbb{P}(W^{N-1} = 0 \,|\, S_1^{N-1} = s_1^{N-1}) \\
&\quad + \mathbb{P}(\Delta y_{N-1} \,|\, S_2^{N-1} = s_1^N, S_1^{N-1} = s_1^{N-1}, W^{N-1} = 1) \\
&\qquad \times \mathbb{P}(S_2^{N-1} = s_1^N \,|\, S_1^{N-1} = s_1^{N-1}) \\
&\qquad \times \mathbb{P}(W^{N-1} = 1 \,|\, S_2^{N-1} = s_1^N, S_1^{N-1} = s_1^{N-1}) \\
&\quad + \sum_{\substack{s_2^{N-1}=1 \\ s_2^{N-1} \neq s_1^{N-1} \\ s_2^{N-1} \neq s_1^N}}^{n} \mathbb{P}(\Delta y_{N-1} \,|\, S_3^{N-1} = s_1^N, S_2^{N-1} = s_2^{N-1}, S_1^{N-1} = s_1^{N-1}, W^{N-1} = 2) \\
&\qquad\qquad \times \mathbb{P}(S_3^{N-1} = s_1^N \,|\, S_2^{N-1} = s_2^{N-1}) \\
&\qquad\qquad \times \mathbb{P}(W^{N-1} \geq 2 \,|\, S_2^{N-1} = s_2^{N-1}, S_1^{N-1} = s_1^{N-1}) \\
&\qquad\qquad \times \mathbb{P}(S_2^{N-1} = s_2^{N-1} \,|\, S_1^{N-1} = s_1^{N-1}),
\end{aligned}$$

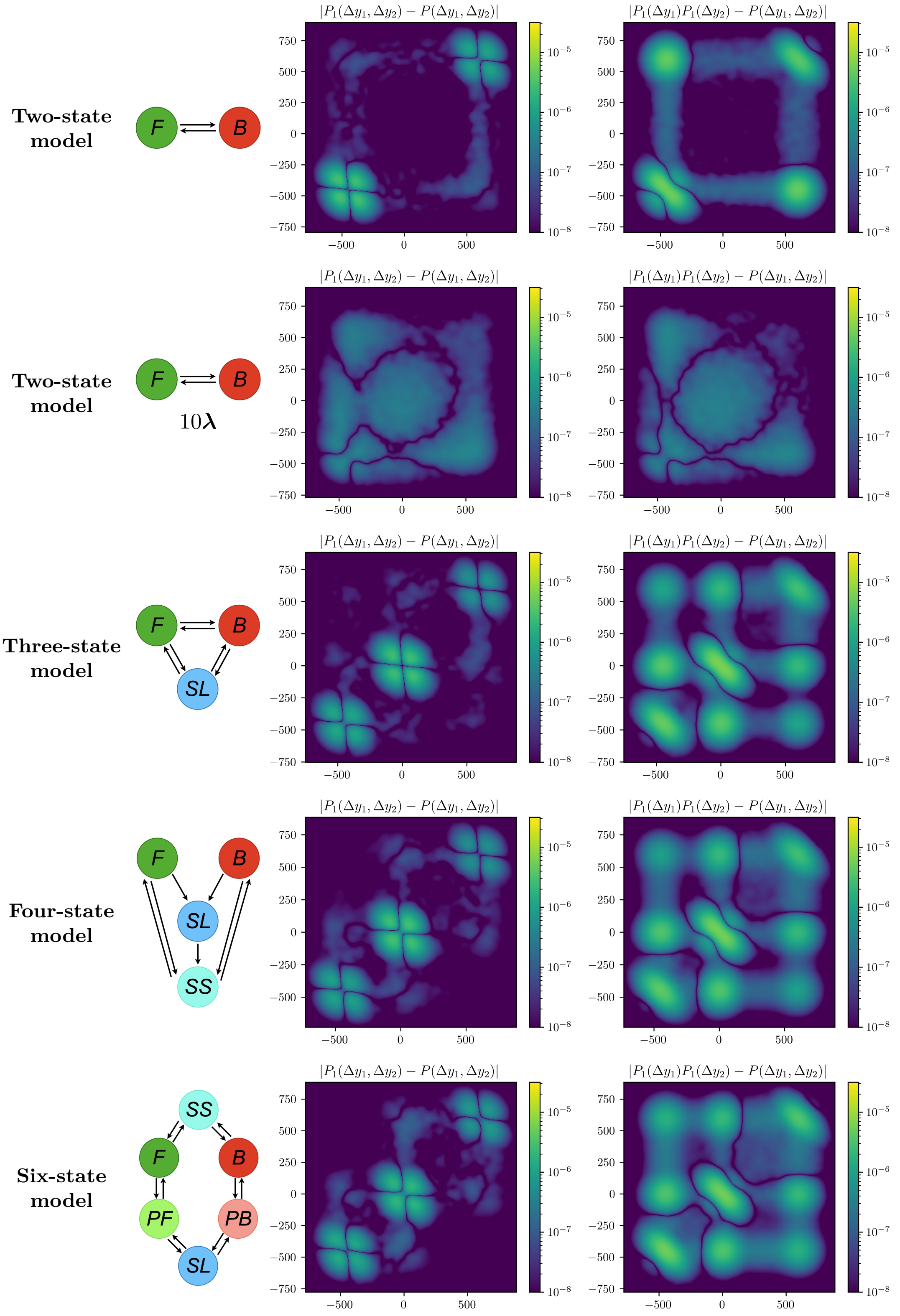

Fig. S5 The absolute value of the error of the approximation of the joint PDF $P_1(\Delta y_1, \Delta y_2)$, defined as $P_1(\Delta y_1, \Delta y_2) - P(\Delta y_1, \Delta y_2)$ where $P(\Delta y_1, \Delta y_2)$ is the empirical PDF for $[\Delta y_1, \Delta y_2]$, is compared with the absolute value of the error of the approximation obtained as the product of the marginals $P_1(\Delta y_1)P_1(\Delta y_2)$, defined as $P_1(\Delta y_1)P_1(\Delta y_2) - P(\Delta y_1, \Delta y_2)$. The panels at the top are obtained using the parameters as specified in the Supplementary Information Figure S1.

for which we can compute all of the terms.

S7 Comparison to the Fokker-Planck equation

The model presented corresponds to the Fokker-Planck equation (or Forward-Kolmogorov equation) in the case with no diffusion (Gardiner, 2009). In particular, the evolution of the probability density function for the exact particle location $p(x, t; s)$ in state $s \in \{1, 2, \ldots, n\}$ and at location x at time $t \geq 0$ and is described by the following equation

$$\frac{\partial p(x, t; s)}{\partial t} + v_s \frac{\partial p(x, t; s)}{\partial x} = \sum_{u=1}^{n} q_{us} p(x, t; u),$$

where q_{us} is the transition rate from state u to state s, and v_s is the velocity in state s. We note that the probability density function for the exact particle location over time is obtained by adding the probability densities over all possible states

$$p(x, t) := \sum_{s=1}^{n} p(x, t; s).$$

References

Ford W (2014) Numerical linear algebra with applications: Using MATLAB. Academic Press, Cambridge, Massachusetts, https://doi.org/10.1016/C2011-0-07533-6

Gardiner C (2009) Markov processes. In: Stochastic Methods. Springer, p 42–76

Kuntz J, Thomas P, Stan GB, et al (2021) Stationary distributions of continuous-time Markov chains: a review of theory and truncation-based approximations. SIAM Review 63(1):3–64. https://doi.org/10.1137/19M1289625

Liggett TM (2010) Continuous time Markov processes: an introduction, vol 113. American Mathematical Society, Providence, Rhode Island, https://doi.org/10.1090/gsm/113

Maday S, Twelvetrees AE, Moughamian AJ, et al (2014) Axonal transport: cargo-specific mechanisms of motility and regulation. Neuron 84(2):292–309. https://doi.org/10.1016/j.neuron.2014.10.019

Norris JR (1998) Markov chains. Cambridge University Press, Cambridge, https://doi.org/10.1017/CBO9780511810633

Ross SM (2014) Introduction to probability models. Academic press, Cambridge, Massachusetts, https://doi.org/10.1016/C2012-0-03564-8
